# Molecular Mechanisms of Hypoxic Responses via Unique Roles of Ras1, Cdc24 and Ptp3 in a Human Fungal Pathogen *Cryptococcus neoformans*

**DOI:** 10.1371/journal.pgen.1004292

**Published:** 2014-04-24

**Authors:** Yun C. Chang, Ami Khanal Lamichhane, H. Martin Garraffo, Peter J. Walter, Maarten Leerkes, Kyung J. Kwon-Chung

**Affiliations:** 1 Molecular Microbiology Section, Laboratory of Clinical Infectious Diseases, NIAID, NIH Bethesda, Maryland, United States of America; 2 Clinical Mass Spectrometry Core, NIDDK, NIH, Bethesda, Maryland, United States of America; 3 Bioinformatics and Computational Biosciences Branch, NIAID, NIH, Bethesda, Maryland, United States of America; Duke University Medical Center, United States of America

## Abstract

*Cryptococcus neoformans* encounters a low oxygen environment when it enters the human host. Here, we show that the conserved Ras1 (a small GTPase) and Cdc24 (the guanine nucleotide exchange factor for Cdc42) play an essential role in cryptococcal growth in hypoxia. Suppressor studies indicate that *PTP3* functions epistatically downstream of both *RAS1* and *CDC24* in regulating hypoxic growth. Ptp3 shares sequence similarity to the family of phosphotyrosine-specific protein phosphatases and the *ptp3Δ* strain failed to grow in 1% O_2_. We demonstrate that *RAS1*, *CDC24* and *PTP3* function in parallel to regulate thermal tolerance but *RAS1* and *CDC24* function linearly in regulating hypoxic growth while *CDC24* and *PTP3* reside in compensatory pathways. The *ras1Δ* and *cdc24Δ* strains ceased to grow at 1% O_2_ and became enlarged but viable single cells. Actin polarization in these cells, however, was normal for up to eight hours after transferring to hypoxic conditions. Double deletions of the genes encoding Rho GTPase Cdc42 and Cdc420, but not of the genes encoding Rac1 and Rac2, caused a slight growth retardation in hypoxia. Furthermore, growth in hypoxia was not affected by the deletion of several central genes functioning in the pathways of cAMP, Hog1, or the two-component like phosphorylation system that are critical in the cryptococcal response to osmotic and genotoxic stresses. Interestingly, although deletion of *HOG1* rescued the hypoxic growth defect of *ras1Δ*, *cdc24Δ*, and *ptp3Δ*, Hog1 was not hyperphosphorylated in these three mutants in hypoxic conditions. RNA sequencing analysis indicated that *RAS1*, *CDC24* and *PTP3* acted upon the expression of genes involved in ergosterol biosynthesis, chromosome organization, RNA processing and protein translation. Moreover, growth of the wild-type strain under low oxygen conditions was affected by sub-inhibitory concentrations of the compounds that inhibit these biological processes, demonstrating the importance of these biological processes in the cryptococcal hypoxia response.

## Author Summary

When *Cryptococcus neoformans*, an environmental fungal pathogen, enters the human host, it encounters a low oxygen condition. The well conserved Ras1 and Cdc24 proteins are known for their key roles in maintenance of the actin cytoskeletal integrity in eukaryotic cells. In this work, we show a unique role of *RAS1* and *CDC24* in the growth of *C. neoformans* in a low oxygen environment. Actin polarization, however, appeared normal in the *ras1Δ* and *cdc24Δ* strains under hypoxic conditions for up to eight hours. We show that *PTP3* is required for hypoxic growth and it can rescue the hypoxic growth defect in *ras1Δ* and *cdc24Δ*. Genetic analysis suggested that *RAS1* and *CDC24* function linearly while *CDC24* and *PTP3* function parallelly in regulating hypoxic growth. RNA sequencing combined with analysis by small molecular inhibitors revealed that *RAS1, CDC24* and *PTP3* regulate several biological processes such as ergosterol biosynthesis, chromosome organization, RNA processing and protein translation which are required in the cryptococcal response to hypoxic conditions.

## Introduction

Oxygen availability is critical for many biochemical reactions in eukaryotic cells and their ability to adapt to oxygen limitation is essential for survival. Most organisms are able to sense the change in environmental oxygen concentration and respond swiftly. For instance, the human fungal pathogen *Cryptococcus neoformans* is an obligate aerobic fungus that grows in an ecological niche with ambient air. Once inhaled by the susceptible host, *C. neoformans* has to adapt to suboptimal levels of oxygen in the lung and disseminates to the brain where oxygen levels are even lower than the lung. How aerobic environmental organisms adapt to suboptimal concentrations of oxygen in the host is an important issue for the understanding of cryptococcal pathogenesis.

Various mechanisms of oxygen sensing and responses to hypoxia in yeast and pathogenic fungi have been reviewed recently [1]–[4]. *C. neoformans* utilizes Sre1, the mammalian sterol regulatory element-binding protein (SREBP) homolog, to control sterol homeostasis, oxygen sensing, and virulence in mice [5], [6]. Sre1 is also the major regulator of the hypoxic response in *Schizosaccharomyces pombe* and it regulates expression of more than 100 genes [7]. However, there are no obvious SREBP orthologs in *Saccharomyces cerevisiae* or in any of the species belonging to the *Candida* clade. These yeast cells respond to limited-oxygen conditions by inducing expression of a large number of hypoxic genes, which encode oxygen-related functions in respiration and biosynthesis of heme, lipids, cell-wall and membranes [2], [4]. Studies using the hypoxia mimetic compound CoCl_2_ revealed that the ability of *C. neoformans* to grow in low oxygen conditions was linked to mitochondrial function, the ability of cells to respond to reactive oxygen species, and gene expression associated with ubiquitination as well as sterol and iron homeostasis [8].

Recent genetic studies have demonstrated that normal actin function and actin-binding proteins are important for the growth of *C. neoformans* in hypoxic conditions [9]. Actin rearrangements are regulated by small GTPases of the Ras and Rho subfamilies [10]. In mammalian cells, Ras transduces signals to multiple pathways that regulate the expression of nuclear genes as well as those required for rearrangement of the actin cytoskeleton [11], [12]. Ras proteins are upstream determinants of actin cytoskeletal integrity and cell stress response as exhibited in many organisms [13]–[15]. Rho-GTPases function as molecular switches by cycling between inactive GPD-bound and active GTP-bound forms. In mammalian cells, the Rho family of G proteins such as Cdc42, Rac, and Rho play complementary roles in the actin cytoskeleton organization [16]. Actin re-polarization at 37°C is delayed in *ras1Δ* mutant cells of *C. neoformans* [17]. Additionally, actin cytoskeletal architecture and cellular morphogenesis are controlled by elements of the Ras pathway in a temperature dependent manner [18], [19]. *C. neoformans* Ras1 also cooperates with conserved Rho-GTPases such as Cdc42 and Rac1 in controlling cell morphology under stressful conditions and regulate filamentous growth during mating [19]–[21]. *C. neoformans* contains two functional Cdc42 paralogs, Cdc42 and Cdc420, which are required for growth at high temperatures but are not required for viability under non-stress conditions [22]. Rac1 and Rac2 function downstream of Ras1 in *C. neoformans* and together with Ste20, which belongs to Cdc42p-activated signal transducing kinase and is a member of the PAK (p21-activated kinase) family, control high-temperature growth and cellular differentiation [20], [23].

Guanine nucleotide exchange factors (GEF), GTPase-activating proteins (GAP) and guanine nucleotide dissociation inhibitors (GDI) are proteins that enable and control the transition of Rho-GTPases between the GPD-bound and active GTP-bound form [24]. In *C. neoformans*, Cdc24, a Cdc42-specific GEF, is a Ras1 effector mediating the ability of this fungus to grow at high temperatures [19]. As in the case of *RAS1*, *CDC24* is required to cause disease. Epistasis and yeast two-hybrid analysis indicated that the Cdc24 homolog of *S. pombe*, Scd1, forms a protein complex with Cdc42 and Ras1 supporting the functional relationship between Ras1 and Cdc24 proteins [25]. In this study, we show that growth of *C. neoformans* in low oxygen conditions requires *RAS1* and *CDC24*. Interestingly, there was no clear evidence that actin polarization was compromised in *ras1Δ* and *cdc24Δ* strains under hypoxic conditions. Suppressor screening enabled the identification of Ptp3 as a downstream effector of Ras1 and Cdc24. *PTP3* encodes a putative homolog of the phosphotyrosine-specific protein phosphatases and shares similarity with the *S. cerevisiae* Ptp3 that regulates the Hog1 mitogen-activated protein kinase [26]. By RNA sequencing analysis and using small molecular inhibitors, we have demonstrated that *RAS1, CDC24* and *PTP3* regulate cryptococcal response to hypoxia that involves several biological processes such as ergosterol biosynthesis, chromosome organization, RNA processing and protein translation.

## Results

### *RAS1* and *CDC24* Are Required for Growth of *C. neoformans* in Hypoxic Conditions

**Figure 1:**
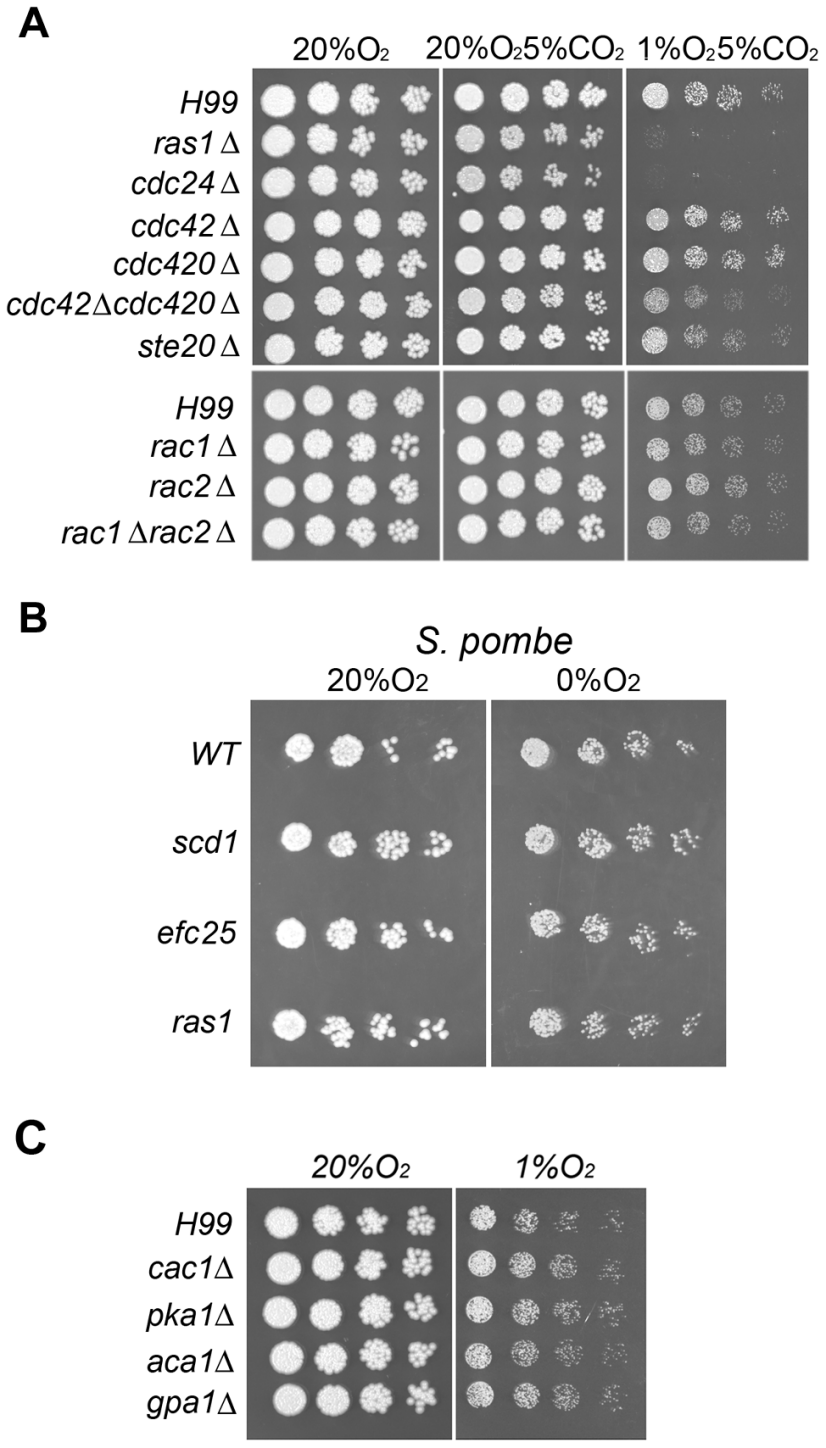
*RAS1* and *CDC24* are required for hypoxic growth in *C. neoformans* but not in *S. pombe*. (A) Three-fold serial dilutions of each strain of *C. neoformans* were spotted on YEPD agar medium and incubated at 20% O_2_, or 1% O_2_ and 5% CO_2_, or 5% CO_2_ and 20% O_2_ at 30°C for 3 days. (B) Cultures of *S. pombe* were spotted on YES medium and incubated at 30°C with 20% O_2_ or with 0% O_2_ for 3 days. (C) The indicated strains were spotted on YEPD agar medium and incubated at 20% O_2_, or 1% O_2_ and 5% CO_2_ at 30°C for 3 days. For convenience, we only indicated the oxygen concentration without mentioning the CO_2_ concentration. The name of each strain is indicated on the left.

It is known that Ras1-Cdc24 signal transduction pathway mediates actin polarization [19] and proper actin function is important for hypoxic response in *C. neoformans* [9]. We, therefore, examined the involvement of genes that function in the Ras1-Cdc24 signal transduction pathway in the cryptococcal response to hypoxia. Figure 1A shows that *ras1Δ* and *cdc24Δ* strains fail to grow at 1% O_2_ but grow comparably to the wild-type H99 in ambient oxygen which suggests an essential role of the Ras1-Cdc24 signaling pathway in hypoxia response. It has been proposed that Ras1 signals utilize multiple Rho-GTPases and Ste20 to coordinately regulate polar growth in *C. neoformans* [22]. We found that deletion of the individual genes encoding the two types of Rho-GTPase genes, including *CDC42*, *CDC420*, *RAC1* and *RAC2*, did not affect the ability of *C. neoformans* to grow in low oxygen. Although growth of the *cdc42Δcdc420Δ* double deletant was slightly reduced in 1% O_2_ compared to the wild-type, growth of the *rac1Δrac2Δ* double deletant was comparable to the wild-type (Figure 1A). Furthermore, deletion of *STE20* only caused a subtle reduction in growth at the same low oxygen condition. *C. neoformans* and *S. pombe* both employ the Sre1 pathway as part of their hypoxia response network suggesting the biological similarity between these two fungi [1]. However, the *S. pombe* mutants, *scd1* (encoding a Cdc24 homolog), *ras1*, and *efc25* (encoding a Ras1-GEF) did not display a hypoxia sensitive phenotype at 0% O_2_ (Figure 1B).

In *C. neoformans*, the Ras1 signaling pathway is known to regulate invasive growth and mating via the cAMP signaling pathway [18], [27]. The cAMP signaling pathway is also involved in response to stress from heavy metals and toxic metabolites [28]. To test the importance of the cAMP stress response pathway in hypoxic conditions, we examined the growth phenotype of mutants defective in the cAMP signaling pathway genes including *gpa1, cac1*, *aca1*, and *pka1*. We found that none of the mutants was susceptible to 1% O_2_ (Figure 1C) which suggested that the *RAS1-CDC24*-dependent hypoxia response is not directly related to the cAMP signaling pathway in *C. neoformans*.

### The *ras1* and *cdc24* Strains Do Not Exhibit a Defect in Actin Polarization under Hypoxic Conditions for up to 8 Hours

**Figure 2:**
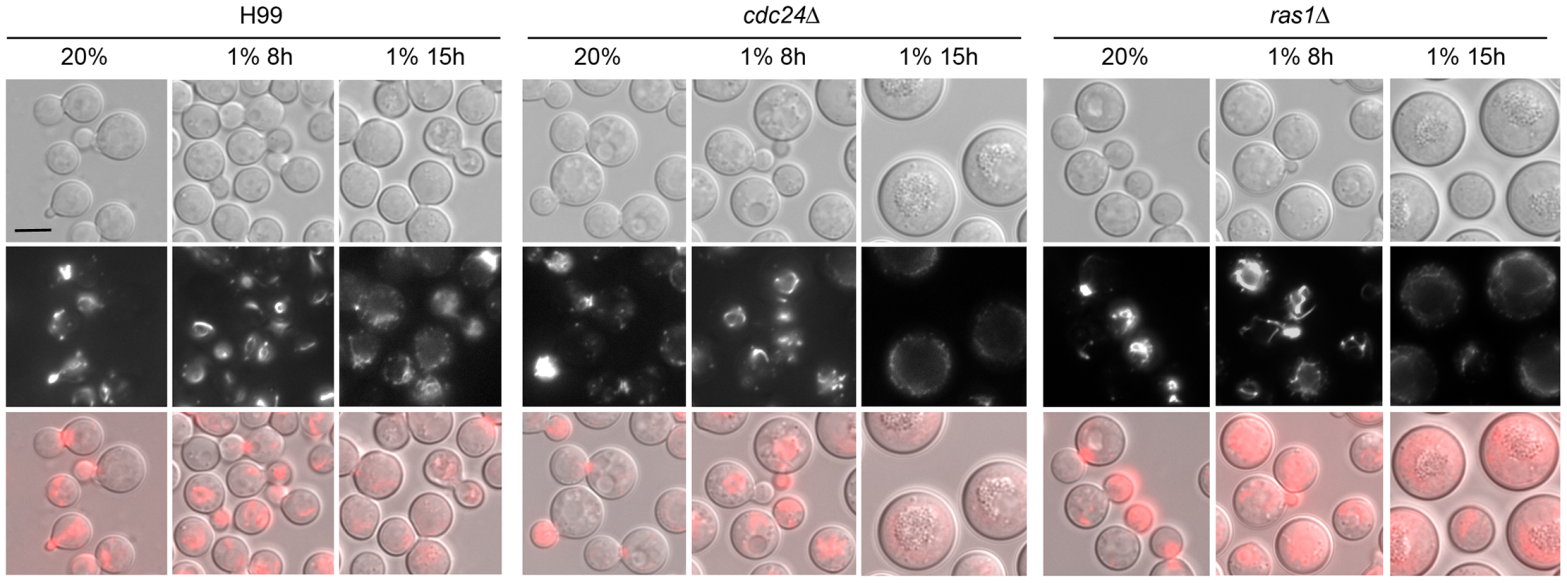
Actin polarization is not clearly influenced by *cdc24Δ* and *ras1Δ* in hypoxic conditions. Each indicated strain was transformed with Lifeact-RFP. The cells were grown to log phase in 20% O_2_ and shifted to 1% O_2_ for 8 h or 15 h at 30°C. The photographs shown are DIC images (top), fluorescent images (middle) and merged fluorescent and DIC images (bottom). Scale bar = 5 µm.

As described previously [17], [19], log phase cells of *ras1Δ* and *cdc24Δ* strains grown in ambient oxygen were slightly larger in size than the wild-type strain (Figure 2). When these cells were shifted to 1% O_2_ and incubated for 8 h, the mutant cells were clearly larger than the wild-type cells (Figure 2). After 15 h at 1% O_2_, both *ras1Δ* and *cdc24Δ* cells ceased to multiply and a large portion of the cells were arrested as large unbudded cells suggesting a compromised budding process. This phenotype is similar to the terminal phenotype of *ras1Δ* and *cdc24Δ* incubated at restricted temperature [17], [19]. Most of the *ras1Δ* and *cdc24Δ* cells, however, were still viable at 1% O_2_ because when these mutants were first incubated at 1% O_2_ for 3 days and then transferred to 20% O_2_ for an additional 3 days, most of the arrested cells at 1% O_2_ resumed growth (Figure S1).

The *ras1Δ* cells manifest depolarized F-actin when grown at restricted temperature and visualized by rhodamine-conjugated phalloidin stain [17]. We examined the distribution of actin by expressing the Lifeact-RFP construct in a *ras1Δ* and a *cdc24Δ* strain. Lifeact contains a 17-amino-acid peptide of Abp140 from *S. cerevisiae* that has been used to visualize actin [29]. Interestingly, no clear difference of Lifeact-RFP signal was observed between the wild-type and either the *ras1Δ* or the *cdc24Δ* strain both at 20% O_2_ or 1% O_2_ for 8 h (Figure 2 middle panels). After 15 h in 1% O_2_, the Lifeact-RFP signal was not clearly visible in the *ras1Δ* and *cdc24Δ* cells due to the lack of budding cells. To quantitatively determine the ability of each strain in polarizing actin to the bud, the percentage of cells containing Lifeact-RFP staining in small to medium sized buds were determined. We observed that actin polarization was similar in wild-type, *ras1Δ* and *cdc24Δ* strains in 20% O_2_ (99%, 100% and 100%, respectively) as well as in 1% O_2_ for 2 h (87%, 87% and 90%, respectively). Similar results were observed in phalloidin stained cells under the same conditions (data not shown). These results indicate that *ras1* and *cdc24* mutations do not visibly affect actin polarization under hypoxic conditions for up to 8 hours.

### Identification of Suppressors for Hypoxic Phenotype of *cdc24Δ*

**Figure 3:**
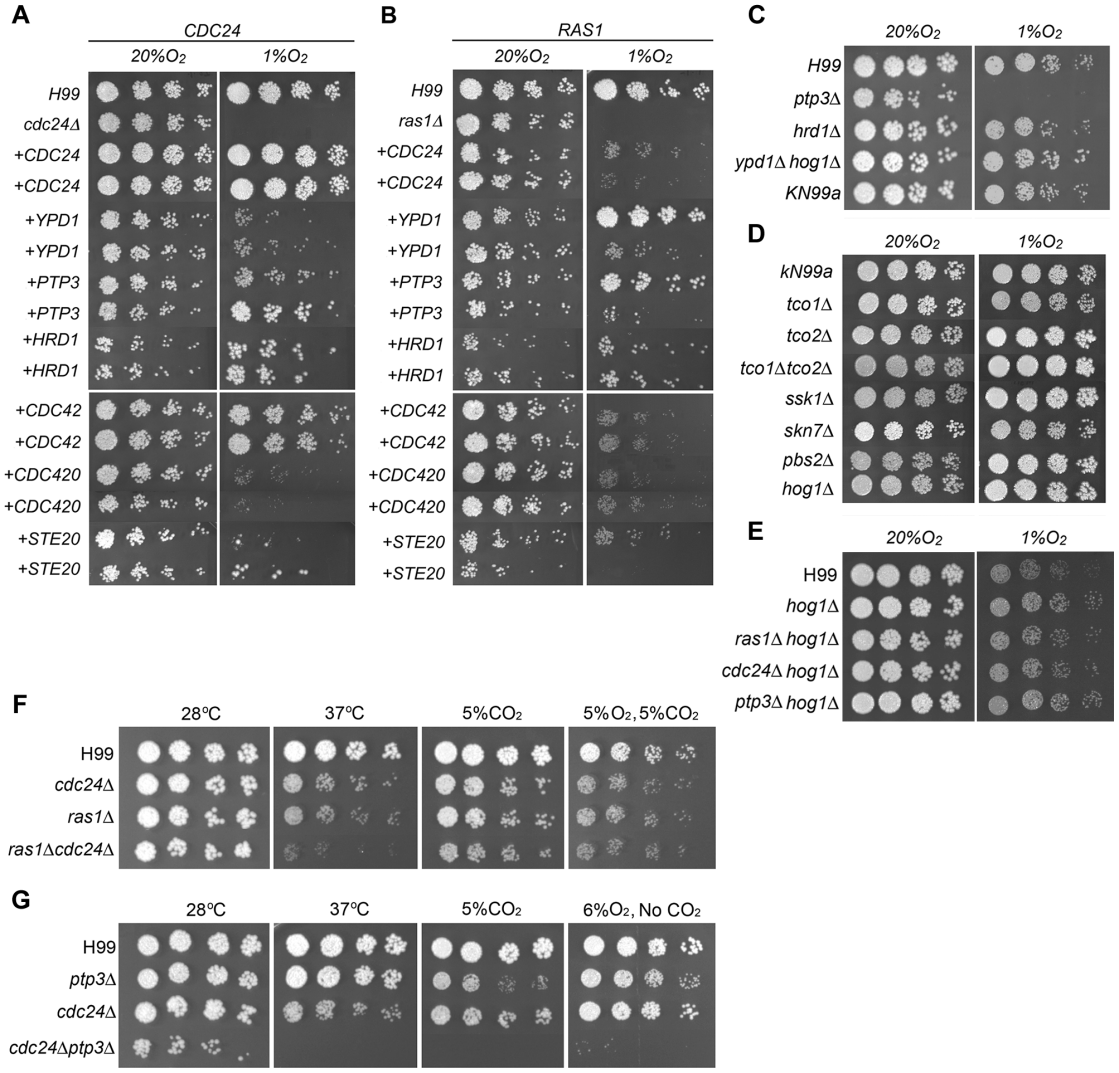
Spot assay for growth phenotype. Three-fold serial dilutions of each strain were spotted on YEPD medium and incubated at indicated conditions. Suppressor test in *cdc24Δ* (A) or *ras1Δ* (B). Two independent transformants of *cdc24Δ* and *ras1Δ* were tested. The cultures were incubated in 20% O_2_ at 30°C for 3 days or in 1% O_2_ at 30°C for 5 days. (C, D and E) Growth phenotype of various mutants. The cultures were incubated in 20% O_2_ at 30°C for 3 days or in 1% O_2_ at 30°C for 3 (C and E) or 5 days (D). Growth assays presented in each panel of the composite images of A–D were performed on the same day. (F) Phenotype of *ras1Δcdc24Δ* double deletant. The cultures were incubated either at 28°C, 37°C, 20% O_2_ with 5% CO_2_ at 28°C, or 5% O_2_ with 5% CO_2_ at 28°C for 3 days, respectively. (G) Phenotype of *cdc24Δptp3Δ* double deletant. The cultures were incubated either at 28°C, 37°C, 20% O_2_ with 5% CO_2_ at 28°C, or 6% O_2_ without CO_2_ at 28°C for 3 days, respectively.

To characterize the Ras1-Cdc24 dependent hypoxic growth pathway, we performed suppressor screens to identify genes whose multicopy presence suppressed the no-growth phenotype of *cdc24Δ* at 1% O_2_. We initially obtained 17 transformants of *cdc24Δ* that were able to grow at 1% O_2_. The inserts in the episomes of these transformants were subsequently PCR-amplified and sequenced. Among the successfully amplified PCR clones, three contained overlapping sequences of *YPD1* (see Table 1 for gene designation of each suppressor and description of the function of *S. cerevisiae* homologs). Three clones contained the entire sequence of *CDC24*, *PTP3*, and *ERJ5* genes, respectively. Two clones each contained *HRD1* and *GEF1* with portions of the 5′ end sequence missing. We cloned the entire genomic regions of *HRD1* and *GEF1* and used to confirm that both *HRD1* and *GEF1* genes suppressed the hypoxia phenotype of *cdc24Δ.* Since *ERJ5* and *GEF1* only weakly restored the phenotype of *cdc24Δ* at 1% O_2_ (Figure S2A), we conducted no further studies on these two genes. Figure 3A shows that extra copies of *CDC24*, *YPD1*, *PTP3* and *HRD1* are able to complement *cdc24Δ* growth defect at 1% O_2_.

**Table 1:** List of genes that suppressed *cdc24Δ* hypoxic phenotype.

Gene name	NCBI gene symbol	Broad Institute locus name	*S. cerevisiae* homolog	SGD description
*CDC24*	CNI03840	CNAG_04243	*CDC24*	Guanine nucleotide exchange factor for Cdc42p; also known as a GEF or GDP-release factor; required for polarity establishment and maintenance, and mutants have morphological defects in bud formation and shmooing; relocalizes from nucleus to cytoplasm upon DNA replication stress
*ERJ5*	CNF02730	CNAG_05700	*ERJ5*	Type I membrane protein with a J domain is required to preserve the folding capacity of the endoplasmic reticulum; loss of the non-essential ERJ5 gene leads to a constitutively induced unfolded protein response
*GEF1*	CNE01370	CNAG_02420	Not found	RhoGEF and PH domains containing protein
*HRD1*	CNH01290	CNAG_05469	*HRD1*	Ubiquitin-protein ligase; required for endoplasmic reticulum-associated degradation (ERAD) of misfolded proteins; genetically linked to the unfolded protein response (UPR); regulated through association with Hrd3p; contains an H2 ring finger; likely plays a general role in targeting proteins that persistently associate with and potentially obstruct the ER-localized translocon
*PTP3*	CNL04680	CNAG_05155	*PTP3*	Phosphotyrosine-specific protein phosphatase involved in the inactivation of mitogen-activated protein kinase (MAPK) during osmolarity sensing; dephosporylates Hog1p MAPK and regulates its localization; localized to the cytoplasm
*YPD1*	CNM01530	CNAG_06151	*YPD1*	Phosphorelay intermediate protein, phosphorylated by the plasma membrane sensor Sln1p in response to osmotic stress and then in turn phosphorylates the response regulators Ssk1p in the cytosol and Skn7p in the nucleus

It has been demonstrated that extra copies of *CDC420* (GenBank ID: DQ991433) and *STE20* suppress the temperature sensitive phenotype of *cdc24Δ* [19]. We introduced extra copies of each of these genes in *cdc24Δ* and examined its growth at 1% O_2_. Figure 3A shows that *CDC42* fully restores the growth of *cdc24Δ* at 1% O_2_ and *STE20* weakly complements the *cdc24Δ* hypoxic phenotype while *CDC420* fails to suppress the phenotype. It has been shown that *CDC24*, *CDC42*, *CDC420*, and *STE20* can suppress the temperature sensitive phenotype of *ras1Δ* [19]. We found that all four of these genes weakly suppress the hypoxic phenotype of *ras1Δ* (Figure 3B). Although *RAC1* could restore thermotolerance in *ras1Δ* mutants [19], *RAC1* and *RAC2* did not suppress the hypoxic phenotypes of *cdc24Δ* or *ras1Δ* (data not shown). Interestingly, three newly identified *cdc24Δ* suppressors, *HRD1*, *PTP3*, and *YPD1* could complement the growth defect of *ras1Δ* at 1% O_2_ (Figure 3B upper panels).

To determine if the newly identified *cdc24Δ* suppressors play a role in cryptococcal growth at 1% O_2_, we deleted *PTP3* and *HRD1* in the wild-type strain H99. Deletion of *PTP3* caused hypersensitivity to 1% O_2_ but not for *hrd1Δ* (Figure 3C). *YPD1* is required for viability of *C. neoformans* and can be only deleted in a background that carries the *hog1* deletion [30]. We examined the phenotype of *ypd1Δhog1Δ* at 1% O_2_ and found that *YPD1* may be not essential for growth in hypoxic conditions (Figure 3C). Furthermore, CnYpd1 is a homolog of a His-containing phosphorelay intermediate protein and the cryptococcal phosphorelay system consists of seven sensor histidine kinases (Tco1 to -7) and two response regulators (Ssk1 and Skn7) [31]. Among the seven sensor histidine kinases, *TCO1* and *TCO2* have been shown to play a role in hypoxia response in a specific strain derived from H99 [6]. However, the deletion of *SSK1*, *SKN7*, *TCO1*, *TCO2*, or *TCO1*/*TCO2* double deletion in KN99a, the mating type **a** derivative of H99, did not affect growth in hypoxic conditions (Figure 3D). This suggests that the cryptococcal phosphorelay system is not essential for response to hypoxia. Additionally, deletion of seven known G protein-coupled receptors (GPCRs) did not affect growth in hypoxic conditions (Figure S2B) suggesting that the pathways linked to GPCRs are not by themselves required for cryptococcal growth in hypoxia.

**Figure 4:**
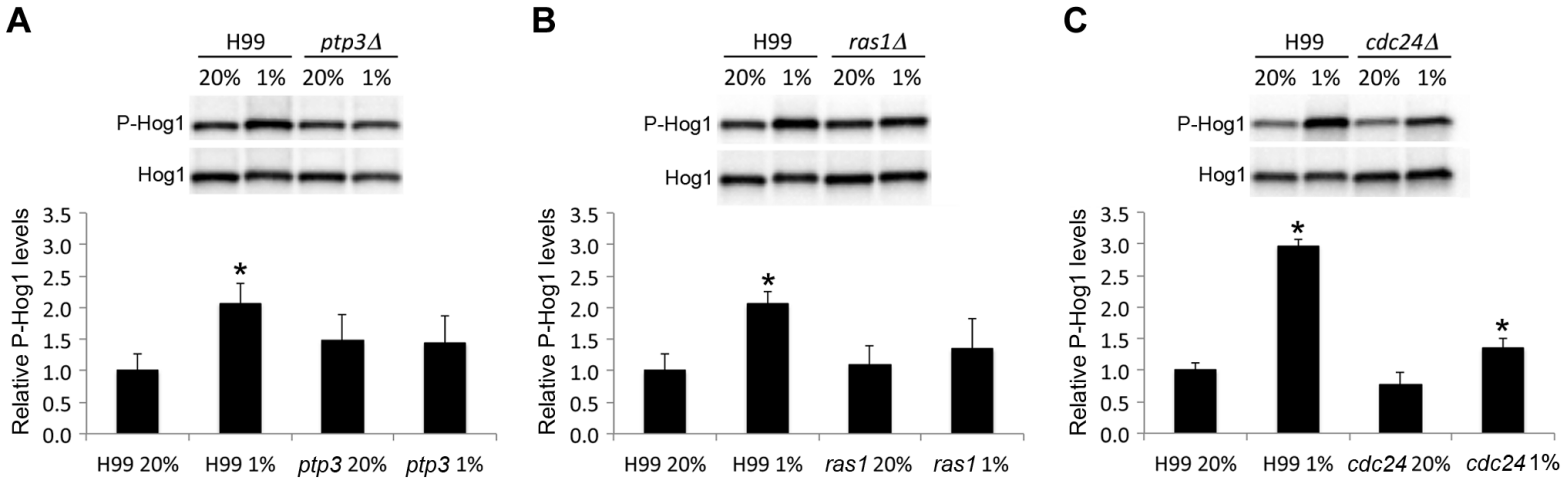
Hog1 is not hyperphosphorylated in *Δptp3Δ*, *ras1Δ*, and *cdc24*. (A, B, and C) Cells of the indicated strains were grown to early logarithmic phase, exposed to 1% O_2_ for 1 hour in YPD medium and total protein extracts were analyzed by Western blot analysis. The phosphorylation status of Hog1 was monitored using anti-phospho-specific p38 antibody (P-Hog1). The same blots were stripped and reacted with anti-Hog1 antibody for Hog1 loading control (Hog1). Levels of Hog1 phosphorylation were quantitated and expressed as relative levels to the control wild-type H99 grown at 20% O_2_. The bar represents standard deviation of four biological repeats. The * indicates p<0.05 compared to H99 at 20% O_2_.

*PTP3* has not been characterized in *C. neoformans*. In *S. cerevisiae*, Ptp3 is involved in the inactivation of mitogen-activated protein kinase (MAPK) during osmolarity sensing and dephosporylation of Hog1 MAPK as well as regulation of its localization [26], [32]–[34]. Moreover, the *S. cerevisiae* Hog1 mediates a hypoxic response in *S. cerevisiae* [35]. To determine the importance of HOG signaling pathway in *C. neoformans* response to hypoxia, we examined the growth of *hog1Δ* and *pbs2Δ*, a MAPK kinase kinase of the HOG signaling pathway [36] at 1% O_2_. Figure 3D shows that although *PTP3* is required for growth in hypoxic conditions, the HOG pathway genes, *HOG1* and *PBS2* are not. Since the cryptococcal Ptp3 may also be a negative regulator of Hog1, as in *S. cerevisiae*, deletion of *PTP3* could cause hyperactivation of Hog1 leading to the inability of *ptp3Δ* to grow in hypoxic conditions. To test this possibility, we deleted *HOG1* in *ptp3Δ.* The *hog1Δ* strain grew better than the wild-type in 1% O_2_ and *ptp3Δhog1Δ* double deletant grew comparable to the wild-type in 1% O_2_ (Figure 3E). We also deleted *HOG1* in *ras1Δ* and *cdc24Δ* strains. Interestingly, both *ras1Δhog1Δ* and *cdc24Δhog1Δ* double deletants grew comparable to the wild-type in 1% O_2_. These data indicate that deletion of *HOG1* compensated for the hypoxic growth defect of *ras1Δ*, *cdc24Δ*, and *ptp3Δ* suggesting existence of genetic interactions between *HOG1* and these three genes. It is possible that cryptococcal Ptp3 regulates the activity of Hog1 by modulating the phosphorylation status of Hog1. Figure 4 shows that Hog1 was phosphorylated at normoxic conditions in H99 as previously described [36]. Interestingly, Hog1 phosphorylation in *ras1Δ*, *cdc24Δ*, and *ptp3Δ* was similar to the wild-type at normoxic conditions. Hog1 phosphorylation significantly increased when cells were transferred to 1% O_2_ for one hour in the wild-type strain but not in the *ptp3Δ* and *cdc24Δ* strains (Figure 4). Although there was a statistically significant increase of Hog1 phosphorylation in *cdc24Δ*, the amount of increase was not as high as in the wild-type. These results demonstrate that Hog1 was not hyperphosphorylated in response to hypoxic treatment in *ras1Δ*, *cdc24Δ*, and *ptp3Δ.*

Suppressor studies in hypoxic conditions suggested that *RAS1* is epistatically upstream of *CDC24*, which is upstream of *PTP3*. To determine if these three genes function linearly or in parallel in regulating hypoxic growth, we generated *ras1Δcdc24Δ* and *cdc24Δptp3Δ* double deletants. The growth of *ras1Δ* and *cdc24Δ* was slightly slower than the wild-type strain at 37°C and *ras1Δcdc24Δ* double deletion strains grew slower than the parental single deletants (Figure 3F). These data indicate that the effect of *RAS1* and *CDC24* double deletions on growth was synthetic at elevated temperatures, suggesting that *RAS1* and *CDC24* may function in compensatory/distinct pathways in regulating thermal tolerance. The growth of *ptp3Δ* was similar to the wild-type at 37°C but the *cdc24Δptp3Δ* double deletant failed to grow at 37°C (Figure 3G). In addition, *PTP3* failed to complement the growth defect of *ras1Δ* or *cdc24Δ* at elevated temperatures (Figure S2). These data suggested that the effect of *CDC24* and *PTP3* double deletions was synthetic at elevated temperatures and *CDC24* and *PTP3* may function in compensatory pathways in regulating thermal tolerance.

Since *ras1Δ*, *cdc24Δ* and *ptp3Δ* could not grow in 1% O_2_, we examined the hypoxic phenotype of double deletions at slightly higher oxygen conditions. The growth of *ras1Δ* and *cdc24Δ* was slower than the wild-type in 5% O_2_ (Figure 3F) and the growth retardation of *ras1Δcdc24Δ* double deletants in 5% O_2_ was no more severe than the parental single deletants. This result suggests that *RAS1* and *CDC24* may function in a linear pathway to handle low oxygen stress. Since *cdc24Δptp3Δ* failed to grow in the presence of 5% CO_2_ (Figure 3G), we examined the hypoxic phenotype of *cdc24Δptp3Δ* in 6% O_2_ without CO_2_ at 28°C. The growth of *ptp3Δ* and *cdc24Δ* was slightly slower than the wild-type in 6% O_2_ without CO_2_ at 28°C. In contrast, *cdc24Δptp3Δ* failed to grow at 6% O_2_ without CO_2_ (Figure 3G) demonstrating that the effect of these double deletions is additive in 6% O_2_ and suggesting that *CDC24* and *PTP3* may function in parallel to manage hypoxic stress.

### Identification of Response Pathways Associated with Hypoxia by RNA Sequencing Analysis

**Figure 5:**
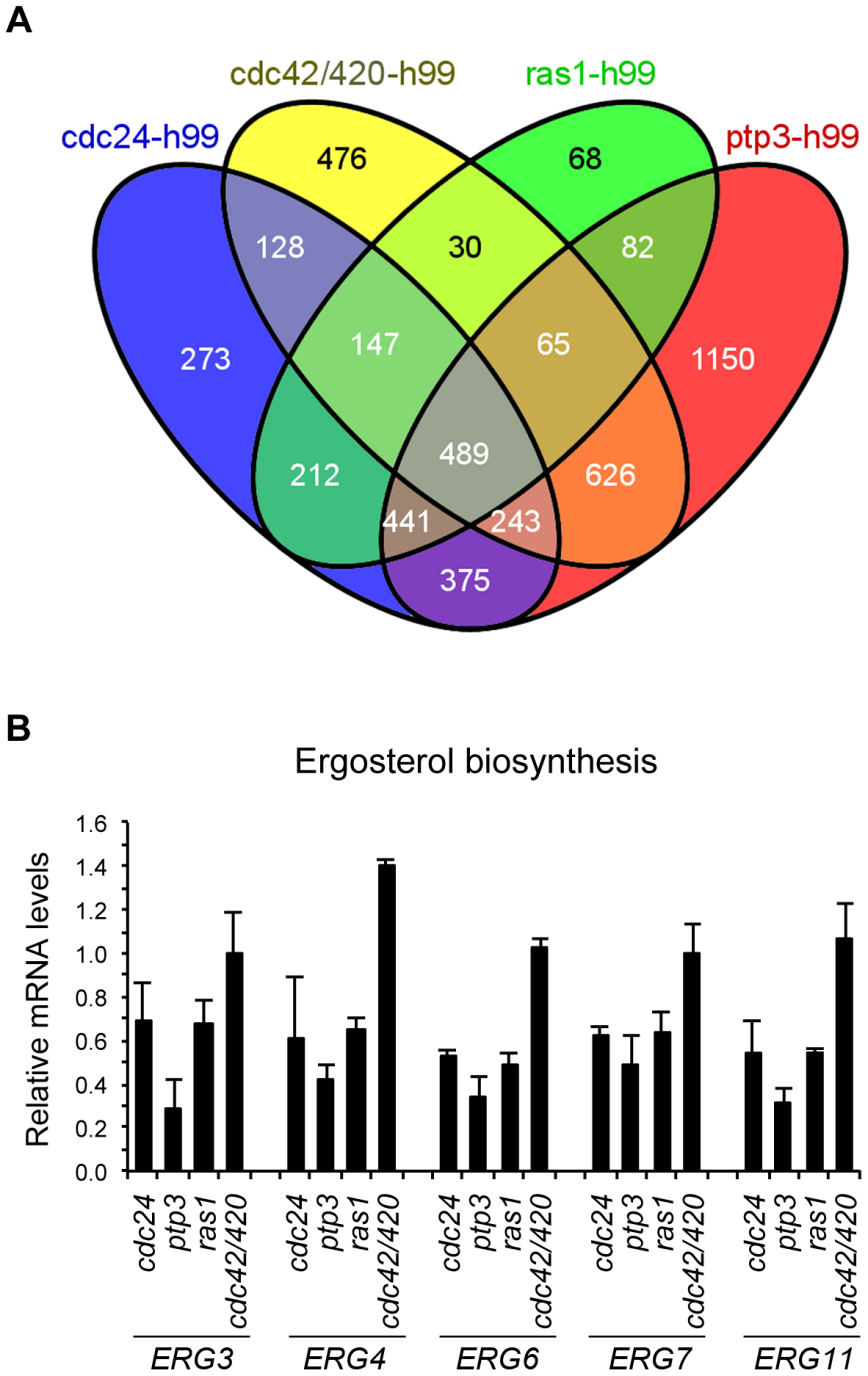
Transcriptome profile analysis. (A) Venn's diagram of the comparison of differential gene expression based on RNA sequencing analysis. Venn's diagram was produced by taking the differentially expressed genes with FPKM cut-off of >10. Each mutant was compared to the wild-type strain H99. The 441 and 489 subsets were selected and analyzed with GO term ontology. (B) Ergosterol biosynthetic genes are down regulated in *cdc24Δ, ptp3Δ* and *ras1Δ*. RNAs were isolated from strains grown in 1% O_2_. Quantitation of the relative transcript levels of each indicated gene was performed by real-time RT-PCR analysis. Data were derived from two biological repeats, normalized with *ACTIN* level and expressed as the amount in each deletant strain relative to that of H99. The error bar represents the range from two biological repeats.

After finding that several common stress response pathways proteins were not individually required for hypoxic response, we used RNA sequencing to uncover the pathways which are involved in cryptococcal response to hypoxic stress. We performed high-throughput sequencing of cDNA made from poly(A) RNAs obtained from 1% O_2_ grown cells of the wild-type, *ras1Δ*, *cdc24Δ*, *ptp3Δ* and *cdc42Δcdc420Δ* double deletant strains. Based on the results of suppressor and double deletants analysis, we speculated that there may be an overlap in the group of genes that are differentially expressed between the wild-type and each mutant of *ras1*, *cdc24* and *ptp3*. We first focused our analysis of RNA sequencing data on the group of genes commonly affected by *ras1Δ*, *cdc24Δ* and *ptp3Δ* but not by *cdc42Δcdc420Δ*, since the *cdc42Δcdc420Δ* double deletant only displayed weak hypoxic phenotype. Among the statistical significance of the differentially expressed genes that had FPKM>10 (fragments per kilobase of transcript per million mapped reads), 441 genes belonging to this group were identified (Figure 5A).

To obtain a global picture of the changes in gene expression, we used DAVID Functional Annotation Tools [37] to search for the over-represented GO terms within this group of genes. We used the tool of functional annotation clustering in DAVID to cluster functionally related annotations into groups for related gene–term relationships [38]. Among the group of 441 genes that showed significant differential expression, we found 4 annotation clusters with an enrichment score greater than 1.4. These included ergosterol biosynthesis, chromosome organization, amino acid biosynthetic process and mRNA processing (Table 2). We used RT-PCR and chose 5 differentially expressed genes from the cluster with the highest enrichment score, ergosterol biosynthetic process, to confirm the expression levels. Figure 5B shows that the expression levels of *ERG3*, *ERG4*, *ERG6*, *ERG7*, and *ERG11* in the ergosterol biosynthesis pathway are consistently lower in *ras1Δ*, *cdc24Δ* and *ptp3Δ* compared to that of the wild-type strain while the expression levels of these gens in *cdc42Δcdc420Δ* double deletant strain are similar or slightly higher than in the wild-type. Therefore, quantitative RT-PCR results support the finding from RNA sequencing analysis.

**Table 2:** Functional annotation clustering of the 441 subset from RNA sequencing results.

Annotation Cluster 1	Enrichment Score: 9.38				
GO Term	Number of genes	*p* Value	Fold Enrichment	Benjamini	FDR
GO:0006694:steroid biosynthetic process	15	2.03E-11	10.8	1.43E-08	3.06E-08
GO:0008204:ergosterol metabolic process	13	4.16E-11	13.2	1.46E-08	6.28E-08
GO:0016129:phytosteroid biosynthetic process	13	4.16E-11	13.2	1.46E-08	6.28E-08
GO:0016128:phytosteroid metabolic process	13	4.16E-11	13.2	1.46E-08	6.28E-08
GO:0006696:ergosterol biosynthetic process	13	4.16E-11	13.2	1.46E-08	6.28E-08
GO:0016126:sterol biosynthetic process	13	2.97E-10	11.5	6.97E-08	4.48E-07
GO:0008202:steroid metabolic process	15	1.33E-09	8.2	2.35E-07	2.01E-06
GO:0016125:sterol metabolic process	13	4.55E-09	9.4	6.41E-07	6.87E-06
GO:0008610:lipid biosynthetic process	20	3.23E-06	3.5	3.79E-04	4.87E-03

Annotation Cluster 2	Enrichment Score: 2.15				
GO Term	Number of genes	*p* Value	Fold Enrichment	Benjamini	FDR
GO:0051276:chromosome organization	24	1.85E-05	2.7	1.86E-03	2.79E-02
GO:0016568:chromatin modification	16	4.87E-05	3.4	4.28E-03	7.35E-02
GO:0006325:chromatin organization	19	1.09E-04	2.8	8.46E-03	1.64E-01

Annotation Cluster 3	Enrichment Score: 1.89				
GO Term	Number of genes	*p* Value	Fold Enrichment	Benjamini	FDR
GO:0008652:cellular amino acid biosynthetic process	14	7.81E-03	2.3	3.69E-01	1.12E+01
GO:0046394:carboxylic acid biosynthetic process	16	8.69E-03	2.1	3.77E-01	1.23E+01
GO:0016053:organic acid biosynthetic process	16	8.69E-03	2.1	3.77E-01	1.23E+01
GO:0009309:amine biosynthetic process	14	1.13E-02	2.2	4.36E-01	1.58E+01
GO:0044271:nitrogen compound biosynthetic process	22	5.35E-02	1.5	7.37E-01	5.64E+01

Annotation Cluster 4	Enrichment Score: 1.40				
GO Term	Number of genes	*p* Value	Fold Enrichment	Benjamini	FDR
GO:0016071:mRNA metabolic process	15	1.21E-02	2.1	4.15E-01	1.68E+01
GO:0000375:RNA splicing, via transesterification reactions	7	2.26E-02	3.1	4.88E-01	2.91E+01
GO:0000377:RNA splicing, via transesterification reactions with bulged adenosine as nucleophile	7	2.26E-02	3.1	4.88E-01	2.91E+01
GO:0000398:nuclear mRNA splicing, via spliceosome	7	2.26E-02	3.1	4.88E-01	2.91E+01
GO:0006397∼mRNA processing	12	3.86E-02	2.0	6.55E-01	4.48E+01

The Gene Ontology cluster analysis was performed using the GO Fat database (GOTERM_BP_FAT), developed as part of the Annotation Tool of the DAVID suite of bioinformatics resources. Enrichment score of 1.3 is equivalent to non-log scale *p* = 0.05 [38]. The clusters with enrichment scores greater than 1.4 and *p* value<0.05 are presented. The number of genes representing each of the GO terms is given with associated *p* value and false discovery rate (FDR).

**Figure 6:**
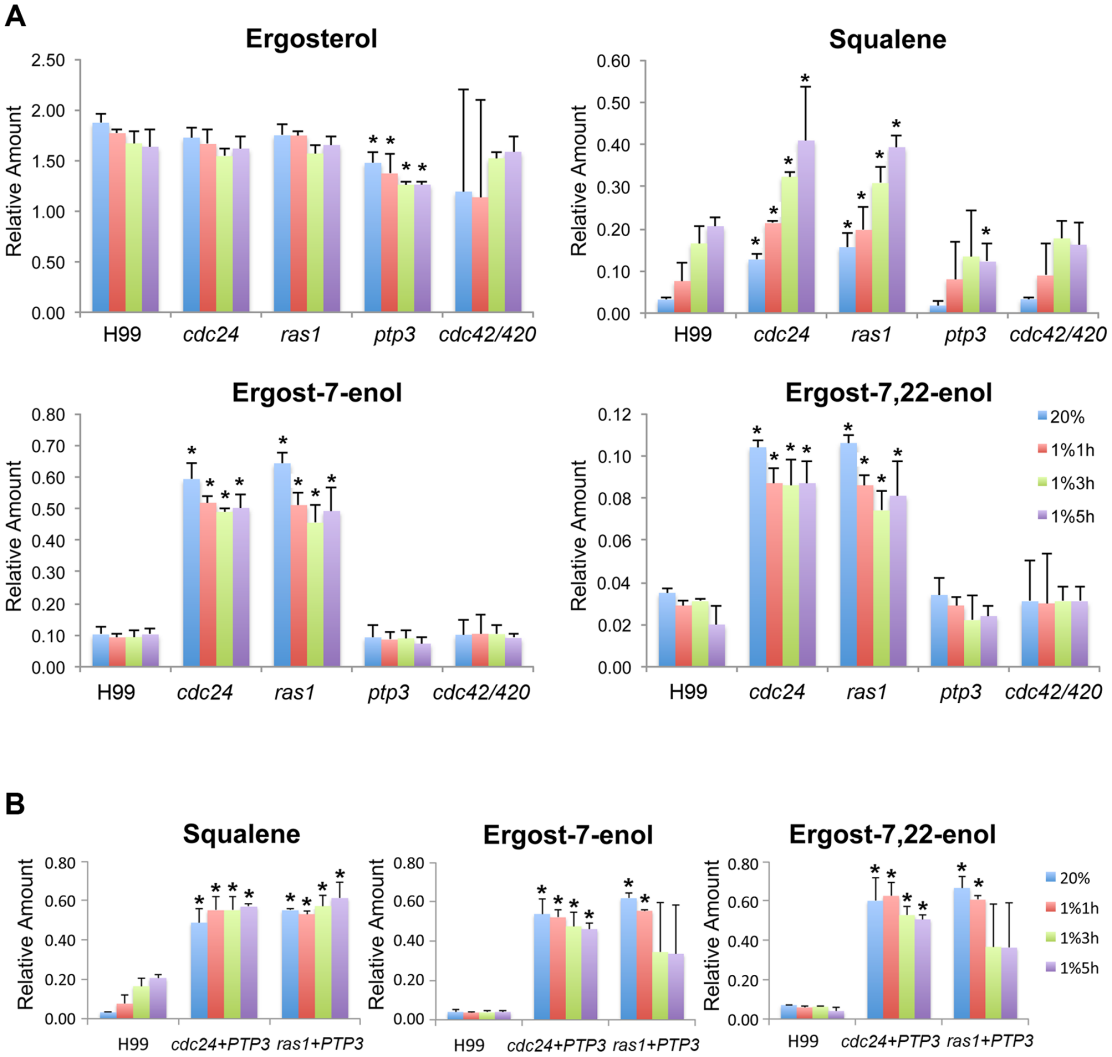
Sterol profile analysis. (A and B) Sterols of each indicated strain were extracted from cells grown in 20% or 1% O_2_ for indicated time points. Sterol profiles were analyzed by gas chromatography/mass spectrometry. The amount of each sterol is expressed as the relative ratio to cholesterol, the internal recovery standard. Only the major ergosterol intermediates (mean relative amount >0.1 according to *cdc24Δ* in 20% O_2_) are shown. The results of discernable minor intermediates (mean relative amount <0.1 according to *cdc24Δ* in 20% O_2_) are shown in Figure S4. Data were derived from three biological repeats. Statistical *t*-test was performed by comparing each mutant from each time point to the corresponding wild-type. The * represents *p*<0.05.

It is likely that the observed changes in transcripts levels for the genes in ergosterol biosynthesis may also reflect the changes in production of ergosterol and its biosynthetic intermediates. We analyzed the sterol profiles of the wild-type and mutants strains to explore this possibility. Cells were transferred from 20% O_2_ to 1% O_2_ and total sterols were extracted and analyzed at several time points after the transfer. We did not find any new ergosterol intermediate in any mutant (Figure S3 and data not shown). Furthermore, no significant difference of ergosterol was detected between the wild-type and all mutants except that *ptp3Δ* produced lower amounts of ergosterol in both normoxic and hypoxic conditions (Figure 6A). Interestingly, almost all the discernible ergosterol intermediates including squalene, eburicol, 4α-methyl fecosterol, ergost-7-enol, and ergost-7, 22-enol accumulated at significantly higher levels in *cdc24Δ* and *ras1Δ* compared to the wild-type in normoxic and hypoxic conditions (Figure 6A and Figure S4). In contrast, the ergosterol intermediates were not significantly different between the wild-type and *ptp3Δ* or *cdc42Δcdc420Δ* except that *ptp3Δ* accumulated significantly lower amounts of squalene and 31-noreburicol after 5 h in 1% O_2_ (Figure 6A and Figure S4). These results indicate that Ras1 and Cdc24 function differently compared to Ptp3 in regulating ergosterol biosynthesis and the production of sterols is similar between *cdc42Δcdc420Δ* and wild-type. Since extra copies of *PTP3* enabled growth of *cdc24Δ* and *ras1Δ* at low oxygen conditions, it is possible that accumulation of the ergosterol intermediates in *cdc24Δ* and *ras1Δ* would be reduced in the *PTP3* over-expressed strains. Interestingly, amounts of the major ergosterol intermediates, squalene, ergost-7-enol, and ergost-7, 22-enol, did not decrease but increased significantly in *cdc24Δ*+*PTP3* and *ras1Δ*+*PTP3* strains at most of the time points (Figure 6B). These data suggest that suppression of the *cdc24Δ* and *ras1Δ* growth deficiency by *PTP3* at 1% oxygen was not due to a reduction in elevated levels of ergosterol intermediates. It also suggests that the accumulation of ergosterol intermediates in *cdc24Δ* and *ras1Δ* may not be the major cause for the inability of these strains to grow in hypoxic conditions.

### Multiple Biological Processes Are Involved in the Cryptococcal Response to Hypoxia

Several biological processes were identified by GO-term analysis using data derived from RNA sequencing. It is possible that the identified processes play a critical role in regulating hypoxic growth in the mutants or the wild-type. Inhibitors known to affect these biological processes were chosen to evaluate their impact on growth under normoxic or hypoxic conditions. The drug concentrations sub-inhibitory to the wild-type strain in normoxic condition were used in all the analyses.

**Figure 7:**
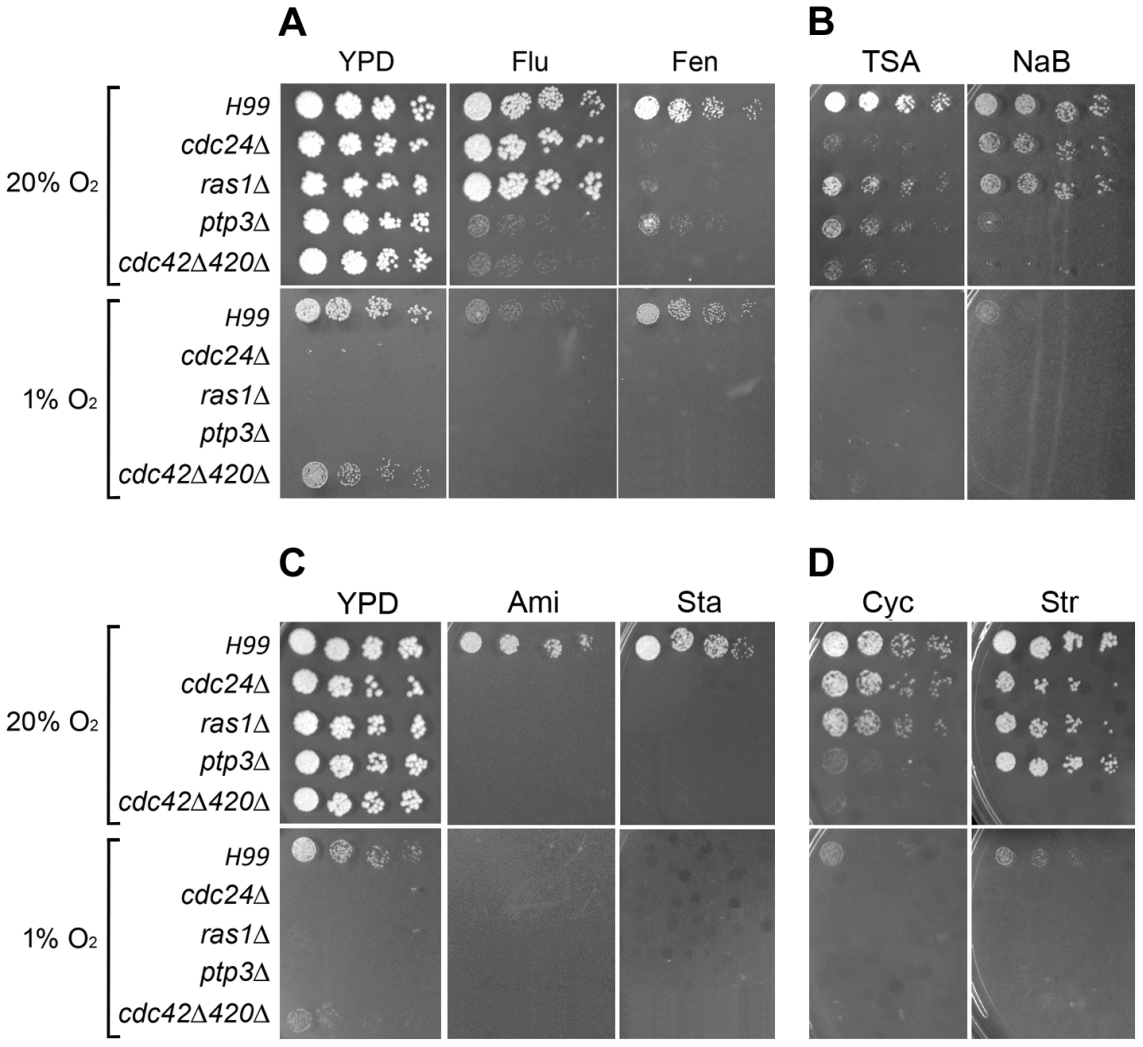
Spot assays to determine the effect of various inhibitors. Cells were serially diluted and spotted onto media containing the indicated inhibitors and incubated at 30°C in 20% CO_2_ for 3 days or in 1% O_2_ at 30°C for 3 days. (A) Ergosterol biosynthesis inhibitors: 8 µg/ml fluconazole (Flu) and 0.25 µg/ml fenpropimorph (Fen). (B) Histone deacetylase inhibitors: 64 µg/ml trichostatin A (TSA) and 128 mM sodium butyrate (NaB). (C) RNA processing inhibitors: 2 mM amiloride hydrochloride hydrate (Ami) and 5 µg/ml staurosporine (Sta). (D) Protein translation inhibitors: 0.1 µg/ml cycloheximide (Cyc) and 40 mg/ml streptomycin (Str).

Ergosterol is an essential component of fungi and its synthesis can be blocked by fluconazole and fenpropimorph. Fluconazole targets the lanosterol 14α-demethylase and fenpropimorph inhibits C14-sterol reductase and C8-sterol isomerase [39]. We selected these two inhibitors to investigate the possible association of ergosterol biosynthesis with the observed phenotype. Figure 7A shows that *ptp3Δ* and *cdc42Δcdc420Δ* were hypersensitive to 8 µg/ml of fluconazole in normoxic conditions compared to the wild-type. In addition, treatment of fenpropimorph resulted in the hypersensitivity of *ras1Δ*, *cdc24Δ*, *ptp3Δ* and *cdc42Δcdc420Δ* in normoxic conditions. The hypersensitivity to ergosterol biosynthesis inhibitors suggested that all the mutants may have a disturbed ergosterol biosynthetic pathway and Ptp3 and Cdc42/Cdc420 may play different roles in this pathway compared to Cdc24 and Ras1. Furthermore, the wild-type strain was hypersensitive to fluconazole in 1% O_2_ but not to fenpropimorph, indicating that inhibition of the lanosterol 14α-demethylase activity in ergosterol synthesis affects the cryptococcal growth in hypoxic conditions.

It is well known that histone acetylation regulates many chromosomal functions. We chose two histone deacetylase inhibitors, trichostatin A (TSA) and sodium butyrate [40] [41] to investigate the possible association of mutant phenotypes with chromosome functions indicated by GO-term analysis. Figure 7B shows that *ras1Δ*, *cdc24Δ*, *ptp3Δ* and *cdc42Δcdc420Δ* double deletant all exhibit hypersensitivity at 64 µg/ml TSA and 128 mM sodium butyrate in normoxic conditions compared to the wild-type, although the level of sensitivity varied among the strains. Furthermore, the wild-type strain was hypersensitive to TSA and sodium butyrate in 1% O_2_ compared to the control YPD medium. To access the possible involvement of mRNA processing, amiloride and staurosporine were selected as inhibitors. Amiloride modulates oncogenic alternative RNA splicing [42] and selectively binds to an abasic site in RNA Duplexes [43]. Although staurosporine is a prototypical ATP-competitive kinase inhibitor that interacts with the ATP binding pocket with little selectivity [44], staurosporine is also known to inhibit yeast RNA splicing *in vitro* by blocking ATP binding to any of the DEXD/H-box RNA helicases involved in splicing [45]. We found that *ras1Δ*, *cdc24Δ*, *ptp3Δ* and *cdc42Δcdc420Δ* double deletant were hypersensitive to staurosporine as well as amiloride in normoxic conditions (Figure 7C). In addition, the wild-type strain grew poorly in the presence of these drugs in 1% O_2_. These results support the notion that chromosome organization and RNA processing are involved in the cryptococcal hypoxic response.

Since *cdc42Δcdc420Δ* displayed slight sensitivity to hypoxic conditions, we also applied DAVID Functional Annotation Tools to analyze the group of 489 genes coordinately expressed among *ras1Δ*, *cdc24Δ*, *ptp3Δ* and *cdc42Δcdc420Δ* (Figure 5A.). Four high enrichment score annotation clusters were identified including ribosome assembly, tRNA amino acylation, regulation of translation and chromosome organization suggesting that protein biosynthesis and/or translation are affected in these mutants (Table 3). We used cycloheximide and streptomycin to investigate the possible association of these genes in protein biosynthesis. Cycloheximide is a glutarimide antibiotic that binds to the 60 Sribosomal subunit and inhibits translation elongation [46]. Streptomycin is an aminoglycoside antibiotic that binds to the small ribosomal subunit of eukaryotic cells and inhibits ribosomal translocation as well as compromises translation fidelity [47]. In comparison to the wild type, growth of *ras1Δ* and *cdc24Δ* was slightly affected in the presence of cycloheximide while the growth of *ptp3Δ* and *cdc42Δcdc420Δ* double deletant was noticeably reduced (Figure 7D). Similarly, the growth of *ras1Δ*, *cdc24Δ*, and *ptp3Δ* was slightly reduced and the growth of *cdc42Δcdc420Δ* double deletant was considerably affected in media containing streptomycin. Furthermore, the wild-type H99 strain grew slower in the presence of streptomycin or cycloheximide in 1% O_2_ compared to the YPD control but not in normoxic conditions suggesting that hypoxic growth requires the fully functional protein translation machinery in *C. neoformans*.

**Table 3:** Functional annotation clustering of the 489 subset from RNA sequencing results.

Annotation Cluster 1	Enrichment Score: 3.15				
GO Term	Number of genes	*p* Value	Fold Enrichment	Benjamini	FDR
GO:0042257:ribosomal subunit assembly	13	5.11E-07	6.2	2.06E-04	7.85E-04
GO:0042255:ribosome assembly	13	6.91E-06	4.9	1.86E-03	1.06E-02
GO:0022618:ribonucleoprotein complex assembly	13	4.17E-05	4.2	8.37E-03	6.40E-02

Annotation Cluster 2	Enrichment Score: 2.15				
GO Term	Number of genes	*p* Value	Fold Enrichment	Benjamini	FDR
GO:0006418:tRNA aminoacylation for protein translation	11	2.07E-03	3.2	1.41E-01	3.13E+00
GO:0043039:tRNA aminoacylation	11	2.07E-03	3.2	1.41E-01	3.13E+00
GO:0043038:amino acid activation	11	2.07E-03	3.2	1.41E-01	3.13E+00
GO:0034660:ncRNA metabolic process	24	3.71E-02	1.5	6.91E-01	4.41E+01

Annotation Cluster 3	Enrichment Score: 2.08				
GO Term	Number of genes	*p* Value	Fold Enrichment	Benjamini	FDR
GO:0006450:regulation of translational fidelity	4	5.5E-04	19.0	6.1E-02	8.4E-01
GO:0006417:regulation of translation	7	4.8E-03	4.3	2.4E-01	7.1E+00
GO:0032268:regulation of cellular protein metabolic process	7	6.6E-03	4.0	2.8E-01	9.6E+00
GO:0010608:posttranscriptional regulation of gene expression	7	6.6E-03	4.0	2.8E-01	9.6E+00
GO:0006448:regulation of translational elongation	4	2.2E-02	6.3	5.7E-01	2.9E+01

Annotation Cluster 4	Enrichment Score: 1.48				
GO Term	Number of genes	*p* Value	Fold Enrichment	Benjamini	FDR
GO:0051276:chromosome organization	24	3.76E-03	1.9	2.09E-01	5.63E+00
GO:0006325:chromatin organization	18	1.56E-02	1.9	5.06E-01	2.15E+01
GO:0016569:covalent chromatin modification	8	3.33E-02	2.6	6.95E-01	4.06E+01
GO:0016570:histone modification	8	3.33E-02	2.6	6.95E-01	4.06E+01
GO:0016568:chromatin modification	13	3.34E-02	1.9	6.81E-01	4.07E+01

The Gene Ontology cluster analysis was performed using the GO Fat database (GOTERM_BP_FAT), developed as part of the Annotation Tool of the DAVID suite of bioinformatics resources. The clusters with enrichment scores greater than 1.4 and showing *p* value<0.05 are presented. The number of genes representing each of the GO terms is given with associated p value and false discovery rate (FDR).

## Discussion

The highly conserved Ras1 and Cdc24 proteins are well known for their importance in the maintenance of actin cytoskeletal integrity. However, the connection between Ras1-Cdc24 signaling pathway and the growth of organisms in a hypoxic environment has not been reported. This study is the first to report on the link of *RAS1* and *CDC24* with hypoxic growth in *C. neoformans*. The fission yeast, *S. pombe* and *C. neoformans* behave similarly in suboptimal levels of oxygen. In *S. pombe*, reduction in sterol synthesis caused by the lack of sufficient oxygen is sensed by Sre1, which is a transcriptional regulator of the hypoxia related genes [7]. However, neither *RAS1* nor *SCD1* (encoding Cdc24 homolog) is required for growth of *S. pombe* in low oxygen conditions (Figure 1). *C. albicans* does not contain a Sre1 homolog but both Ras1 and the adenylate cyclase Cdc35 are known to be associated with hyphal growth in hypoxic conditions [48]. Unlike the *ras1Δ* in *C. neoformans*, however, the *C. albicans ras1Δ* and *cdc35Δ* strains are able to grow in yeast form under hypoxic conditions. Thus, the involvement of Ras1-Cdc24 signaling pathway in hypoxic growth is not a universal fungal paradigm.

Which pathways do Ras1 and Cdc24 proteins utilize to regulate hypoxic growth in *C. neoformans*? Our data excludes a few possible candidates. First, although actin function is required for proper growth in hypoxic conditions [9], reorganization of the actin cytoskeleton did not appear to be different in *ras1Δ* and *cdc24Δ* strains compared to the wild-type strains for up to 8 hours. However, extra copies of *CDC42* suppressed the growth defect of *cdc24Δ* and *ras1Δ* under hypoxia (Figure 3). It is possible that Cdc42 regulates processes other than actin polarization or that the failure of *cdc24Δ* and *ras1Δ* to proliferate in hypoxic conditions is in part due to otherwise unnoticeable malfunction in actin polarization. Second, genes from pathways involved in osmotic and genotoxic stress such as cAMP signaling, Hog1, and the two-components like phosphorelay, are not individually required for growth of *C. neoformans* in hypoxic conditions. These results parallel the observation that the Ras1-Cdc24 signaling pathway operates differently from pathways that are employed in response to osmotic and genotoxic stresses [28]. Furthermore, seven known G protein-coupled receptors (GPCRs) were not required for growth in hypoxic conditions suggesting that the cryptococcal cells do not utilize these receptors to sense hypoxic stress. Thus, Ras1 and Cdc24 proteins play an unusual role(s) in *C. neoformans* to overcome hypoxic stress.

We have noted that although growth of the *cdc42Δcdc420Δ* double deletant was only slightly susceptible to 1% O_2_, it was hypersensitive to all the tested inhibitors even in the normoxic conditions. Cdc42 and Cdc420 belong to the Rho-GTPase family and the Rho-GTPases in all eukaryotic cells are key regulators of the signaling pathways that control actin organization and morphogenetic processes (see recent reviews [49], [50]). Unlike the case in *S. pombe* and *S. cerevisiae* [51], [52], *CDC42* was not essential in *C. neoformans* and the *cdc42Δcdc420Δ* double deletant strain was viable under normal growth conditions [22]. Hypersensitivity to all the tested inhibitors may be due to inhibition of growth in otherwise sick strains or, alternatively, it substantiates the importance of Cdc42 type Rho-GTPase for basic biological functions in *C. neoformans*.

It has been shown that a single Rho GTPase is regulated by more than one RhoGEF or RhoGAP [53]. At the same time, RhoGEFs and RhoGAPs regulate more than one Rho protein. In *C. neoformans*, the Rho GTPase genes *CDC42*, *CDC420* and *RAC1* can restore thermotolerance of the *ras1Δ* mutants while only *CDC420* visibly suppresses the temperature sensitive phenotype of *cdc24Δ* [19]. We have observed that only *CDC42* can restore the ability of *cdc24Δ* to grow at 1% O_2_ while *CDC42* and *CDC420* weakly suppress the hypoxic phenotype of *ras1Δ*. In addition, *RAC1* and *RAC2* did not suppress the hypoxic phenotypes of *cdc24Δ* or *ras1Δ*. Hence, the paralogs of these Rho-GTPases play different roles under different environmental conditions in *C. neoformans*. Additionally, we observed that single mutants of Rho-like GTPases did not impair growth in hypoxic conditions. It is possible that there is enough functional redundancy among the related Rho-like GTPases that single mutants (or even double mutants) may not display a strong phenotype in low oxygen. This possibility was to a certain extent supported by the reduced growth of *cdc42Δcdc420Δ* double deletant strain in 1% O_2_ but not by the *rac1Δrac2Δ* double deletant strain. The growth defect of *cdc42Δcdc420Δ*, however, was not as severe as *ras1Δ* or *cdc24Δ* at 1% O_2_ suggesting that other Rho-GTPases may be involved. Furthermore, triple deletants *cdc42Δcdc420Δrac1Δ* and *cdc42Δcdc420Δrac2Δ* grew comparably to the *cdc42Δcdc420Δ* double deletant strains in 1% O_2_ (data not shown). It is possible that the hypoxic growth phenotype of *cdc42Δcdc420Δrac1Δrac2Δ* quadruple deletant may provide more information but we have so far been unable to generate such a mutant. Alternatively, it is also possible that other types of Rho-GTPases may be involved in the regulation of growth in hypoxia. The detailed mechanisms of how Rho-GTPases and their regulators contribute to the hypoxia response remain to be elucidated.

Among the hypoxic mutants of *C. neoformans* thus far identified [5], [8], [9], [54], [55], *ras1Δ* and *cdc24Δ* displayed the clearest phenotype in 1% O_2_ and enabled us to perform suppressor screening. It is clear that 1% O_2_ or elevated temperature did not kill *ras1Δ* and *cdc24Δ* cells since they were able to resume growth once transferred back to ambient conditions (Figure S1). It is not clear, however, what the physiological state of *ras1Δ* and *cdc24Δ* cells is under hypoxic conditions. Because *cdc24Δ* and *rasΔ1* behaved similarly in accumulation of ergosterol biosynthetic intermediates and sensitivity to many inhibitors, which was different from *ptp3Δ*, it is likely that the pathways controlled by Ptp3 are different from Ras1 and Cdc24. The genetic interactions among *RAS1*, *CDC24* and *PTP3* are complicated. *PTP3* was able to suppress the hypoxic growth phenotype but not thermal intolerance of *cdc24Δ* and *ras1Δ*. *CDC24* was able to suppress the hypoxic and thermal intolerance phenotype of *ras1Δ* but *RAS1* could not suppress the hypoxic phenotype of *ptp3Δ* and *cdc24Δ* (Figure 3 and data not shown) [19]. Growth analysis of double mutants at elevated temperature suggests that *RAS1*, *CDC24* and *PTP3* may function in parallel to regulate thermal tolerance. In contrast, under hypoxic conditions *RAS1* and *CDC24* may function in a linear pathway to regulate hypoxic growth while *CDC24* and *PTP3* may function in parallel for such growth. Details of the mechanism as to how *RAS1*, *CDC24* and *PTP3* interact require further study.

Ptp3 shares similarity to the family of phosphotyrosine-specific protein phosphatases that are important in cell signaling [56], [57]. Oxidation of tyrosine phosphatases in hypoxia followed by re-oxygenation functionally links the tyrosine phosphatases to the hypoxia response in mammalian systems [58]. However, the involvement of Ptp3 in hypoxic response in lower eukaryotes has not been established. It is known that Hog1 in *S. cerevisiae* mediates the hypoxic response [35] and disruption of *PTP3* in *S. cerevisiae* results in constitutive Hog1 tyrosine phosphorylation [26]. Therefore, it is possible that the deletion of cryptococcal *PTP3* causes hyperactivation or constitutive activation of Hog1 that leads to the inability of *ptp3Δ* to grow in hypoxic conditions and deletion of *HOG1* in *ptp3Δ* eliminates the Hog1 influence. However, we showed that deletion of cryptococcal *PTP3* did not result in the elevation of Hog1 phosphorylation in normoxic conditions and Hog1 phosphorylation in hypoxic conditions was much lower in *ptp3Δ* compared to the wild-type strain. Thus, the inability of the *ptp3Δ* strain to grow in hypoxic conditions is not due to hyperactivation of Hog1. These results indicate that the interaction between Ptp3 and Hog1 is different between *C. neoformans* and *S. cerevisiae*. Interestingly, *hog1Δ* grew better than the wild-type in 1% O_2_ and *ptp3Δhog1Δ*, *ras1Δhog1Δ* and *cdc24Δhog1Δ* double deletants also grew as well as the wild-type in 1% O_2_. It is possible that the enhanced growth of *hog1Δ* somehow compensates for the growth deficiency of *ptp3Δ*, *ras1Δ* and *cdc24Δ* in hypoxic conditions thereby enabling the double deletants to grow in 1% O_2_. The detailed mechanisms of interactions between *HOG1* and *PTP3*, *RAS1*, or *CDC24* are yet to be elucidated.

We employed RNA sequencing to elucidate the putative pathways that are affected by mutations in *RAS1*, *CDC24*, and *PTP3*. Based on the results of the GO term functional clustering analysis, we identified several pathways that are commonly affected by these mutants that were verified by phenotypic studies using small molecular inhibitors. Hypersensitivity of the mutants to inhibitory compounds has been used extensively as a tool to dissect the gene function. Such approaches may have a disadvantage in that targets of some of the drugs may not be specific. For example staurosporine is a broad range kinase inhibitor and the observed phenotype may not be due to targeted effect. To partially circumvent such a caveat, two different inhibitors were chosen for each examined pathway. Hypersensitivity of the mutants to fluconazole and fenpropimorph suggests the association of Ras1, Cdc24 and Ptp3 in the ergosterol biosynthesis pathway. Time course analysis of sterol profiles confirms that ergosterol biosynthesis is indeed affected in these mutants. Nevertheless, correlation between the amounts of the ergosterol intermediates and transcript levels of the four selected genes involved in ergosterol biosynthesis pathway was not apparent. It is possible that higher amounts of ergosterol intermediates occurring in the stressed *ras1Δ* and *cdc24Δ* mutants resulted in a compensatory reduction in transcript levels of the four selected *ERG* genes. At least six ergosterol intermediates were accumulated to higher levels in *ras1Δ* and *cdc24Δ*, but not in *ptp3Δ*. In contrast, *ptp3Δ* produced lower amounts of ergosterol compared to the wild type while the amount of ergosterol did not change in *ras1Δ* and *cdc24Δ* suggesting that Ptp3 functions differently in regulating ergosterol biosynthesis compared to Ras1 and Cdc24. Furthermore, it has been shown that deletion of the gene encoding sterol regulatory element-binding protein, *SRE1*, causes a reduction of growth in hypoxic conditions [5], [6]. The *sre1* mutants also displayed a decrease in ergosterol content and increase in several ergosterol intermediates but the patterns of the changes are different from the results of our mutants. Therefore, it is likely that the mechanism regulated by Sre1 is different form Ras1, Cdc24 and Ptp3.

Inhibitors studies show that *ras1Δ*, *cdc24Δ* and *ptp3Δ* all exhibited various degrees of hypersensitivity to inhibitors of chromosome organization, RNA processing and protein translation in a normoxic condition. Although it is possible that the hypersensitivity phenotype is due to inhibition of growth in otherwise already defective strains, it is more likely that these biological processes are perturbed in these mutants. The retarded growth of the wild-type strain in the presence of these drugs under hypoxia apparently suggest that *C. neoformans* requires these biological processes to be fully functional in the management of stress generated by hypoxic conditions. Post-translational modification of histone proteins is known to play a critical role in regulating chromatin structure (for a review see [59]). A recent study has demonstrated that the *S. pombe* histone H2A dioxygenase Ofd2 regulates gene expression during growth in hypoxia, which suggests that chromosome organization is important to the hypoxia response [60]. However, the exact modification of H2A by Ofd2 remains to be determined. We have also isolated a cryptococcal *asc1* mutant that displayed a hypoxic phenotype (data not shown). Asc1 of *C. neoformans* shares similarity to *S. cerevisiae* Asc1 and mammalian RACK1, which is a conserved core component of the eukaryotic ribosome and functions in translational control [61]. Furthermore, previous functional analysis of *C. neoformans* mutants showed that the ability to respond to reactive oxygen species and mitochondrial function are important for growth under CoCl_2_ and low oxygen conditions [8]. Taken together, it is clear that *C. neoformans* overcomes hypoxic stress by employing many fundamental biological processes that had not been implicated in other organisms.

## Materials and Methods

### Strains, Media and Growth Conditions

All strains used in this study were derived from the genome sequence strain H99 and are listed in Table S1. YEPD contains 1% yeast extract, 2% Bacto-peptone and 2% dextrose. YES medium contains 0.5% yeast extract, 3% glucose and 225 µg/ml each of uracil, adenine, leucine, histidine, and lysine. Low-oxygen conditions (5% CO_2_ and 1% O_2_) were maintained using an Invivo2 400 workstation at the indicated temperatures (Ruskinn, UK). For simplicity, we only mention the oxygen concentration without describing the CO_2_ concentration unless specified in our study. The GasPakPlus anaerobic system (BRL) was used to generate 0% O_2_ conditions. Spot assay was conducted by serial dilutions of cultures, spotted on agar plates, incubated at conditions as indicated for 3–6 days.

### Microscopy

Zeiss Axiovert fluorescent microscope equipped with an AxioCam MRm digital camera was used to visualize fluorescent and differential interference contrast microscopy (DIC) images. Axiovision (version 4.0) was used to capture the images that were further processed by Adobe Photoshop CS4 software. Percentage of the polarized cells was determined as described [13].

### Screening of *cdc24Δ* Suppressors

*cdc24Δ* was transformed with a multicopy suppressor library made in a multicopy episomic plasmid, pYCC725, from LP2, a B-3501 derived strain [62]. Suppressor clones were selected by plating the transformants on a medium containing 100 µg/ml nourseothricin at 1% O_2_ and 28°C. Three separate screens were performed and 17 transformants were isolated. The plasmids in transformants were isolated and reconfirmed for their ability to restore the growth of *cdc24Δ* at 1% O_2_. The inserts in the episomes of the transformants were PCR-amplified using primers OYC725B and OYC725C and the amplified PCR fragments were sequenced as described [62]. The genomic content in the PCR amplified region was identified by a BLAST search of the B-3501 genome sequence. Episomal plasmids from each clone were rescued in *E. coli* and retransformed into the *cdc24Δ* strain to confirm the phenotype. For *HRD1* and *GEF1*, the entire gene region was cloned by PCR and reconfirmed for its ability to suppress the hypoxia phenotype of *cdc24Δ*.

### Gene Deletion and Complementation

Gene deletion was carried out via homologous recombination by biolistic transformation [63]. Overlapping PCR technique was used to generate deletion constructs [64]. PCR and Southern hybridization was used to confirm homologous integrations. Wild type genes were PCR amplified from the strain H99, cloned and sequenced as described [9]. To generate complementation construct, each gene was either inserted in the multiple cloning site of pYCC744 which contained the NAT gene as a selectable marker [9] or cloned by overlapping PCR with Hygromycin gene as selectable maker.

### Protein Extraction and Immunoblot Analysis

Cells were grown in YPD medium to early log phase in normoxic conditions, spun down and transferred to 1% O_2_ for 1 hr. Equal volume of ice-cold stop buffer (0.9% NaCl, 1 mM NaN_3_, 10 mM Na-EDTA, 50 mM NaF) was added to the culture. Cells were spun down, washed once in ice-cold stop buffer and lyophilized. Lyophilized cells were disrupted with 1-mm zirconia/silica bead using FastPrep-24 (MP Biomedicals, CA) and resuspended in lysis buffer as described [36]. Cell lysates were spun down at 4°C and protein concentrations were determined using Bio-Rad Bradford Protein assay reagent (Richmond, CA). An equal amount of protein (20 µg) was loaded on the Any kD Criterion TGX Stain-Free gel (Bio-Rad, Richmond, CA) and proteins were transferred to a PVDF membrane. The western blot was incubated with a rabbit phospho-p38-MAPK antibody (Cell Signaling, Beverly, MA) and with a secondary anti-rabbit horseradish peroxidase–conjugated antibody. The blot was developed using the Clarity Western ECL (Bio-Rad, Richmond, CA). The signal was quantitated using ChemiDoc MP imaging system (Bio-Rad, Richmond, CA). The blot was stripped and used for detection of Hog1 with a rabbit polyclonal anti-Hog1 antibody (Santa Cruz Biotechnology, Santa Cruz, CA). The ratio of the signal intensity between phosphorylated Hog1 and total Hog1 was calculated and expressed as relative phosphorylation levels of the wild-type control.

### RNA Sequencing

To isolate RNA from 1% O_2_ grown cells, overnight cultures of wild-type and each deletant strain were refreshed in fresh YPD media for 5 h in ambient air at 28°C and shifted to 1% O_2_ for 2 h at 28°C. RNA was extracted from cryptococcal cells using Trizol (Invitrogen, Carlsbad, CA) and purified with RNeasy MinElute cleanup kit (Qiagen, Valencia, CA). The RNAseq was performed at RML Research Technologies Section, NIAID, NIH. The Illumina TruSeq RNA Sample Preparation Kit (Illumina, San Diego, CA) and its workflow were used for the preparation of barcoded RNA-Seq libraries. Final library products were quantified by qPCR using a KAPA Illumina GA Library Quantification Kit (KAPA Biosystems, Boston, MA) and sequenced on a HiSeq 2000 (Illumina) to produce paired 100 bp reads. Each of the 12 RNAseq libraries were given a unique barcode and pooled for clustering. We used 4 lanes on the HiSeq for sequencing, each lane containing the 12 libraries. Initial processing was performed using Illumina pipeline (CASAVA 1.8). TruSeq adapters were then trimmed and the reads were quality filtered with the FastXToolkit. All reads were mapped, using TopHat vl. 3.0 [65], to the *C. neoformans* H99 reference genome provided by the Broad Institute. The total number of sequence reads ranged from 78 to 102 million pairs of which nearly 87.6%–88.9% were uniquely aligned and properly paired to the reference genome assembly. The mapped reads were then used downstream by Cufflinks software packages [65] for transcript assembly, differential expression, and for gene model construction for RNA-Seq. A union of transcripts from all four samples was produced by Cuffcompare from individually assembled transcripts of each sample which yielded 6967 transcripts. Cuffdiff was used to determine statistically significant differences between strains on assembled transcripts. Gene expression was normalized using the number of fragments per kilobase of transcript per million mapped reads (FPKM). Venn's diagram was produced by taking the statistically significant differentially expressed genes with FPKM cut-off of >10. Gene ontology analysis was performed using DAVID Functional Annotation Tools and with the tool of functional annotation clustering and GO Fat database (GOTERM_BP_FAT) [37]. Based on the homology between H99 and JEC21, each of the H99 gene identification designated by Broad institute was converted to the JEC21 gene identification before using DAVID since DAVID did not recognize the Broad institute identification. The analyzed RNA-Seq data has been submitted to the Sequence Read Archive (SRA) at NCBI and can be viewed under accession number of experiment SRX347687.

### Sterol Analysis

Log phase grown cells were spun down and transferred to 1% O_2_ for 1, 3 or 5 hrs or 20% O_2_ for 2 hrs in YPD medium. Cells were harvested for sterol extraction. Sterol extraction and analysis was carried out as described [31]. Briefly, 10 mM of sodium azide was added to the culture medium immediately before harvest. Cells (1×10^8^ total) were harvested by centrifugation and resuspended in 9 ml methanol and 4.5 ml 60% (wt/vol) KOH together with 5 µg cholesterol which was used as an internal recovery standard. Samples were heated to 75°C in a water bath for 2 h to complete the saponification and the sterols were then extracted with hexane. The extracted sterols were analyzed and characterized by GC-MS (gas chromatography-mass spectrometry) using an ISQ mass spectrometer from Thermo Electron, coupled to a Trace GC Ultra chromatograph, from Thermo Electron, in the EI (electron impact ionization) mode. This GC-MS instrument used a Restek 5MS fused silica column (30 m length, 0.25 mm i.d., 25 µm film thickness) with a program temperature from 200°C (1 min) to 300°C at a rate of 10°C/min. Cholesterol and ergosterol were identified by comparison with standards. The mass spectral data used in combination with a database (NIST/EPA/NIH Mass Spectral Library version 2.0) and published spectra from *C. neoformans* [66] allowed the identification of several peaks for which there were no standards available. The nomenclature of the discernable ergosterol intermediates was followed according to the *C. neoformans* study [66]. Relative amounts of each ergosterol intermediate were determined by comparing the area under the peaks in the chromatogram versus the area under the cholesterol peak to correct for recovery.

### Preparation and Analysis of Nucleic Acid

Isolation and analysis of genomic DNA was carried out as described previously [67]. RNA was treated with RNAse-free DNAse (Ambion, Austin, TX) to remove genomic DNA before quantitative real time reverse transcription PCR (qRT-PCR). cDNA was synthesized using high-capacity cDNA archive kit (Applied Biosystems, Foster City, CA). The qRT-PCR was performed using 20 µl triplicate reactions with SYBR select master mix from two biological replicates and the ABI PRISM 7500 sequence detection system (Applied Biosystems, Foster City, CA). The PCR efficiency and CT determination was performed using the algorithm as described [68]. The primers used for RT-PCR are listed in Table S2. Data were normalized with *ACTIN1* level and expressed as the amount in each deletant strain relative to that of H99.

## Supporting Information

Figure S1 Hypoxic conditions or elevated temperature do not kill *ras1Δ* and *cdc24Δ*. Three-fold serial dilutions of each strain were spotted on YPD plates and incubated in 20% O_2_ or 1% O_2_ for 3 days at 28°C or 20% O_2_ at 39°C for 3 days. After 3 days, the plate from the 1% O_2_ at 28°C and the plate from 39°C were transferred to 20% O_2_ at 28°C for additional 3 days. Picture shows that *ras1Δ* and *cdc24Δ* resume growth after 1% O_2_ or 39°C treatment.
(TIF)

Figure S2 (A) *ERJ5* and *GEF1* weakly complement the hypoxia phenotype of *cdc24Δ. cdc24Δ* was transformed with *ERJ5* or *GEF1*. Cells of indicated strains were spotted on YEPD agar medium and incubated at 20% O_2_ or 1% O_2_ and 5% CO_2_ at 30°C for 3 days. (B) Deletion of seven known GPCRs does not affect growth in hypoxic conditions. Cells of indicated strains were spotted on YEPD agar medium and incubated at 20% O_2_ or 1% O_2_ and 5% CO_2_ at 30°C for 3 days. (C) *ras1Δ and cdc24Δ* suppressors fail to complement the thermal tolerance defect. *ras1Δ* (left) and *cdc24Δ* (right) were transformed with indicated suppressors respectively. Cells of indicated strains were spotted on YEPD agar medium and incubated at 30°C or 39°C.
(TIF)

Figure S3 GC analysis of the sterol profile. (A, B and C) Total sterols were extracted from indicated strains grown at 1% O_2_ and analyzed by GC-MS. The identity of GC peaks relevant to the study is indicated. Cholesterol was used as an internal recovery standard. The sterol profiles of *ptp3Δ* and *cdc42Δcdc420Δ* were similar to H99 (data not shown).
(TIF)

Figure S4 Sterol profile analysis. Sterols of cells from each strain grown in 20% or 1% O_2_ at indicated times were extracted and analyzed by gas chromatography/mass spectrometry. The amount of each sterol is expressed as the relative ratio to cholesterol, the internal recovery standard. The results of discernable minor intermediates (mean relative amount <0.1 according to *cdc24Δ* in 20% O_2_) are shown. Data were derived from three biological repeats. Statistical *t*-test was performed by comparing each mutant from each time point to the corresponding wild-type. The * represents *p*<0.05.
(TIF)

Table S1 List of strains relevant to this study.
(DOC)

Table S2 Primers used for RT-PCR.
(DOC)
