# Supplementary figures and images for: Molecular Mechanisms of Hypoxic Responses via Unique Roles of Ras1, Cdc24 and Ptp3 in a Human Fungal Pathogen Cryptococcus neoformans
Source: PLoS Genet. 2014 Apr 24;10(4):e1004292. doi: 10.1371/journal.pgen.1004292 (PMC3998916; doi:10.1371/journal.pgen.1004292)

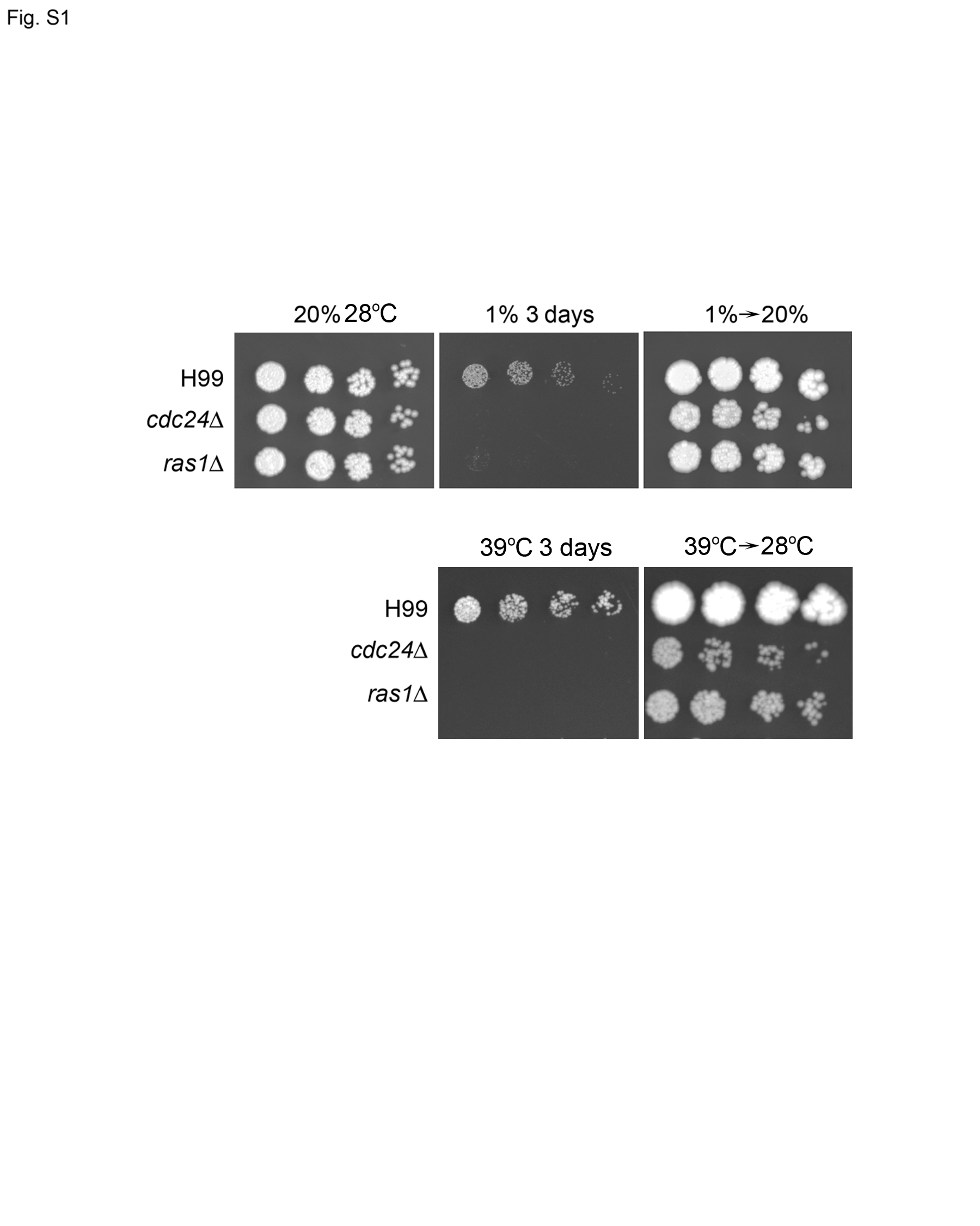

Supplement: Figure S1 — Hypoxic conditions or elevated temperature do not kill ras1Δ and cdc24Δ. Three-fold serial dilutions of each strain were spotted on YPD plates and incubated in 20% O2 or 1% O2 for 3 days at 28°C or 20% O2 at 39°C for 3 days. After 3 days, the plate from the 1% O2 at 28°C and the plate from 39°C were transferred to 20% O2 at 28°C for additional 3 days. Picture shows that ras1Δ and cdc24Δ resume growth after 1% O2 or 39°C treatment. (TIF) [file pgen.1004292.s001.tif]

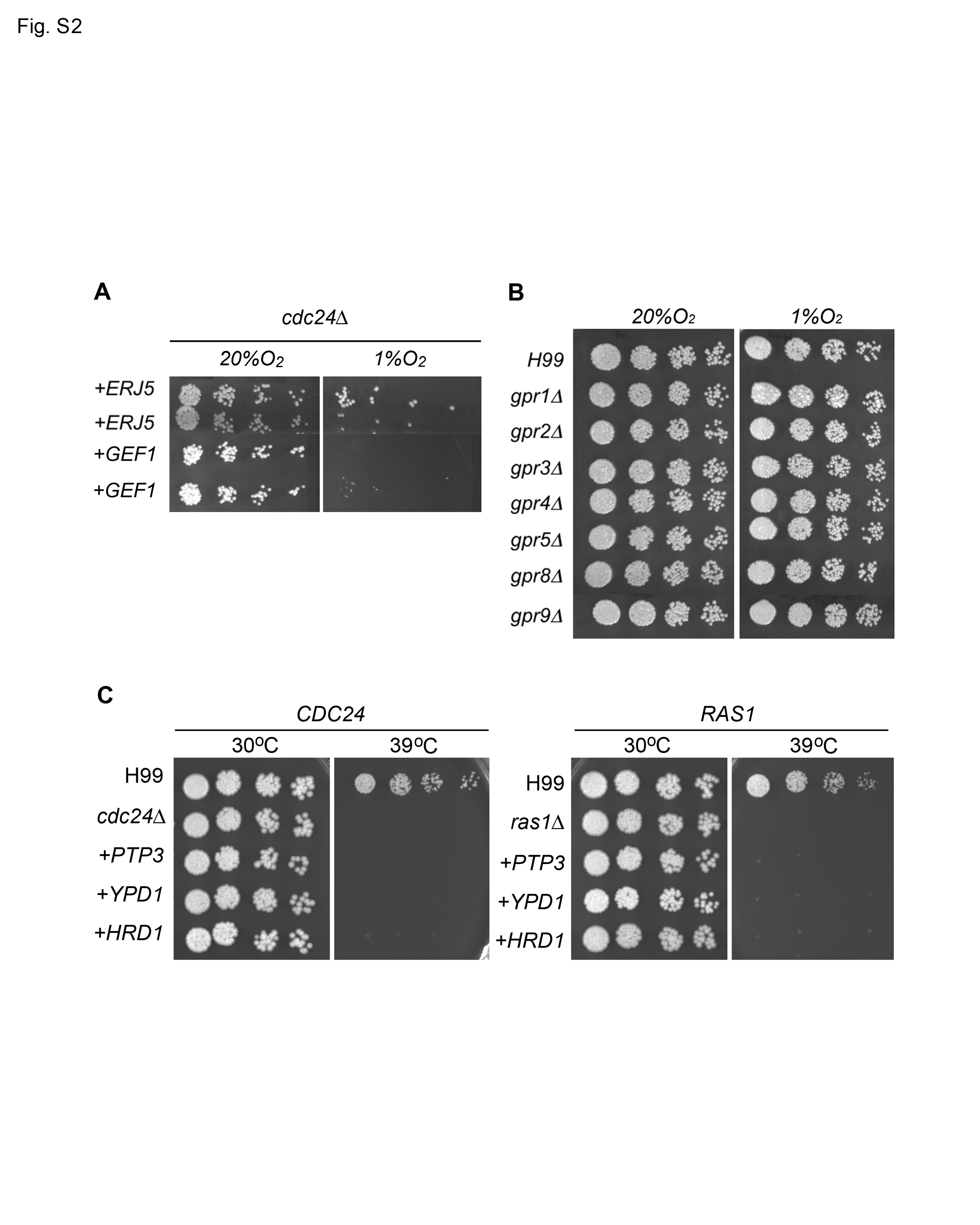

Supplement: Figure S2 — (A) ERJ5 and GEF1 weakly complement the hypoxia phenotype of cdc24Δ. cdc24Δ was transformed with ERJ5 or GEF1. Cells of indicated strains were spotted on YEPD agar medium and incubated at 20% O2 or 1% O2 and 5% CO2 at 30°C for 3 days. (B) Deletion of seven known GPCRs does not affect growth in hypoxic conditions. Cells of indicated strains were spotted on YEPD agar medium and incubated at 20% O2 or 1% O2 and 5% CO2 at 30°C for 3 days. (C) ras1Δ and cdc24Δ suppressors fail to complement the thermal tolerance defect. ras1Δ (left) and cdc24Δ (right) were transformed with indicated suppressors respectively. Cells of indicated strains were spotted on YEPD agar medium and incubated at 30°C or 39°C. (TIF) [file pgen.1004292.s002.tif]

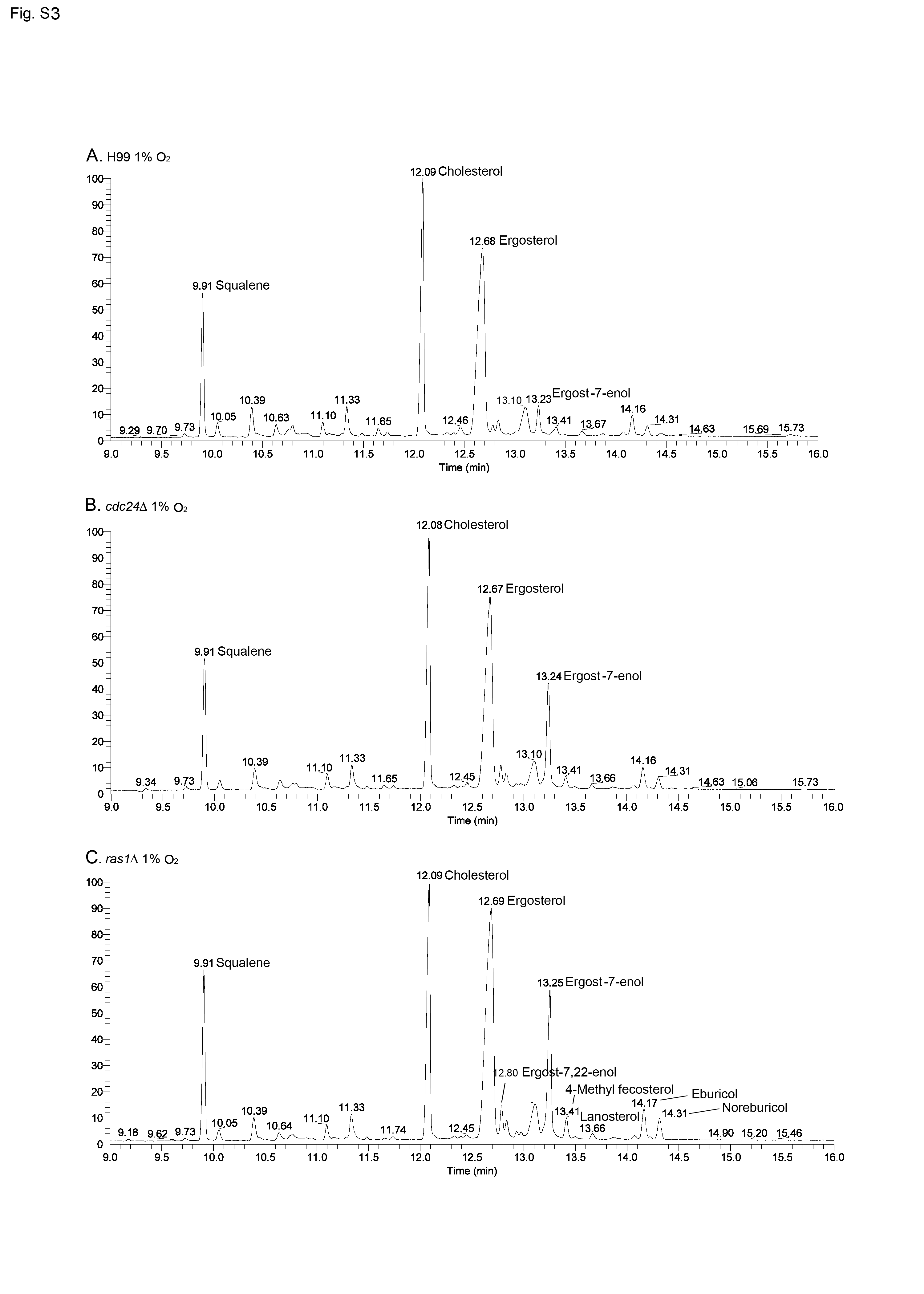

Supplement: Figure S3 — GC analysis of the sterol profile. (A, B and C) Total sterols were extracted from indicated strains grown at 1% O2 and analyzed by GC-MS. The identity of GC peaks relevant to the study is indicated. Cholesterol was used as an internal recovery standard. The sterol profiles of ptp3Δ and cdc42Δcdc420Δ were similar to H99 (data not shown). (TIF) [file pgen.1004292.s003.tif]

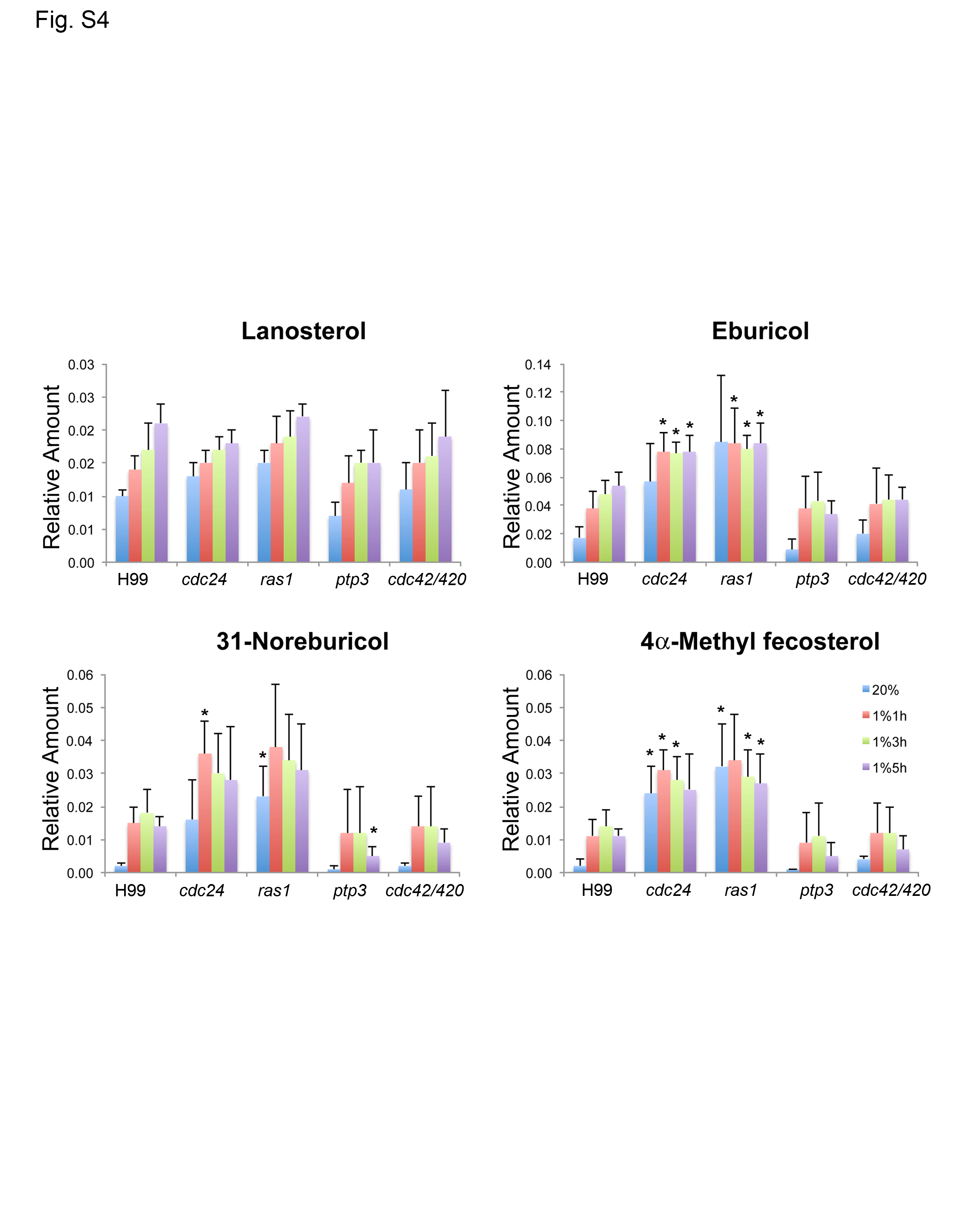

Supplement: Figure S4 — Sterol profile analysis. Sterols of cells from each strain grown in 20% or 1% O2 at indicated times were extracted and analyzed by gas chromatography/mass spectrometry. The amount of each sterol is expressed as the relative ratio to cholesterol, the internal recovery standard. The results of discernable minor intermediates (mean relative amount <0.1 according to cdc24Δ in 20% O2) are shown. Data were derived from three biological repeats. Statistical t-test was performed by comparing each mutant from each time point to the corresponding wild-type. The * represents p<0.05. (TIF) [file pgen.1004292.s004.tif]
