# Supplementary material for: Molecular Mechanisms of Hypoxic Responses via Unique Roles of Ras1, Cdc24 and Ptp3 in a Human Fungal Pathogen Cryptococcus neoformans
Source: PLoS Genet. 2014 Apr 24;10(4):e1004292. doi: 10.1371/journal.pgen.1004292 (PMC3998916; doi:10.1371/journal.pgen.1004292)
Supplement: Table S1 — List of strains relevant to this study. (DOC) [file pgen.1004292.s005.doc]

**Table S1.** List of strains relevant to this study.

| Strains | Descriptions | | Source | |  |
| --- | --- | --- | --- | --- | --- |
| H99 | wild type; *MATα* | |  | |  |
| C1597 | *MATα cdc24∆::NEO Lifeact-RFP::HYG* | | This study | |  |
| C1599 | *MATα Lifeact-RFP::HYG* | | This study | |  |
| C1601 | *MATα ras1∆::NEO Lifeact-RFP::HYG* | | This study | |  |
| C1603 | *MATα ptp3∆::NEO* | | This study | |  |
| C1605 | *MATα aca1∆::NEO* | | This study | |  |
| C1630 | *MATα ptp3∆::NEO PTP3::HYG* | | This study | |  |
| C1639 | *MATα hrd1∆::NEO* | | This study | |  |
| C1666 | *MATα rac1∆::HYG rac2∆::NEO* | | This study | |  |
| C1670 | | *MATα ras1∆::NEO cdc24::HYG* | | This study | |
| C1673 | | *MATα cdc24::HYG ptp3∆::NEO* | | This study | |
| C1674 | | *MATα ptp3∆::NEO hog1∆::HYG* | | This study | |
| C1680 | | *MATα rac1∆::NEO* | | This study | |
| C1682 | | *MATα ras1∆::NEO hog1∆::HYG* | | This study | |
| C1684 | | *MATα cdc24∆::NEO hog1∆::HYG* | | This study | |
| CDX1 | *MATα gpr1∆::NAT* | | C. Xue | |  |
| CDX3 | *MATα gpr2∆::NEO* | | C. Xue | |  |
| CDX5 | *MATα gpr3∆::NEO* | | C. Xue | |  |
| CDX6 | *MATα gpr4∆::NEO* | | C. Xue | |  |
| CDX11 | *MATα gpr5∆::NAT* | | C. Xue | |  |
| CDX14 | *MATα gpr8∆::NAT* | | C. Xue | |  |
| CDX16 | *MATα gpr9∆::NAT* | | C. Xue | |  |
| CBN20 | *MATα ste20∆::NEO* | | A. Alspaugh | |  |
| CBN32 | *MATα cdc24∆::NEO* | | A. Alspaugh | |  |
| CBN45 | *MATα ras1∆::NEO* | | A. Alspaugh | |  |
| ERB002 | *MATα cdc42∆::NAT* | | A. Alspaugh | |  |
| ERB005 | *MATα cdc420∆::NAT* | | A. Alspaugh | |  |
| ERB011 | *MATα cdc42∆::NAT cdc420::NEO* | | A. Alspaugh | |  |
| ERB032 | *MATα rac2∆::NEO* | | A. Alspaugh | |  |
| YSB42 | *MATα cac1∆::NAT* | | Y. Bahn | |  |
| YSB64 | *MATα hog1∆::NEO* | | Y. Bahn | |  |
| YSB81 | *MATa hog1∆::NEO* | | Y. Bahn | |  |
| YSB83 | *MATa gpa1∆::NAT* | | Y. Bahn | |  |
| YSB125 | *MATa pbs2∆::NEO* | | Y. Bahn | |  |
| YSB188 | *MATα pka1∆::NAT* | | Y. Bahn | |  |
| YSB321 | *MATa tco1∆::NEO* | | Y. Bahn | |  |
| YSB324 | *MATα tco1∆::NAT-STM#102 tco2::NEO* | | Y. Bahn | |  |
| YSB412 | *MATa tco2∆::NAT-STM#116* | | Y. Bahn | |  |
| YSB429 | *MATa ssk1∆::NAT-STM#205* | | Y. Bahn | |  |
| YSB434 | *MATa skn7∆::NAT-STM#201* | | Y. Bahn | |  |
| YSB780 | *MATa hog1∆ ypd1∆::NAT-STM#242* | | Y. Bahn | |  |
| ED666 | *S. pombe h^+^, leu1-32,ura4-D18,ade6-6-M210* | | P. Espenshade | |  |
| V2-08-A2 | *S. pombe h^+^, leu1-32,ura4-D18,ade6-6-M210 efc25* | | P. Espenshade | |  |
| V2-10-G3 | *S. pombe h^+^, leu1-32,ura4-D18,ade6-6-M210 scd1* | | P. Espenshade | |  |
| V2-20-F1 | *S. pombe h^+^, leu1-32,ura4-D18,ade6-6-M210 ras1* | | P. Espenshade | |  |
