# Supplementary material for: Molecular Mechanisms of Hypoxic Responses via Unique Roles of Ras1, Cdc24 and Ptp3 in a Human Fungal Pathogen Cryptococcus neoformans
Source: PLoS Genet. 2014 Apr 24;10(4):e1004292. doi: 10.1371/journal.pgen.1004292 (PMC3998916; doi:10.1371/journal.pgen.1004292)
Supplement: Table S2 — Primers used for RT-PCR. (DOC) [file pgen.1004292.s006.doc]

**Table S2.** Primers used for RT-PCR.

| Oligo name | Oligo sequence (5' to 3') |
| --- | --- |
| erg3f | ACCTACCACCCGTCTATTGCA |
| erg3r | GGGATATCGATTGGCGAATG |
| erg4f | CCCGACTTACATCCAAACCAA |
| erg4r | CGCGAGCCCCCAGAA |
| erg6f | ATCTGGCCGACCGAGATG |
| erg6r | GCCTTCCAGATGTCACCTTCA |
| erg7f | GGGTGCTTGGCCGTTCT |
| erg7r | GAGGCCCTCGGCTGTACAG |
